# Invasive breast cancer and breast cancer mortality after ductal carcinoma in situ in women attending for breast screening in England, 1988-2014: population based observational cohort study

**DOI:** 10.1136/bmj.m1570

**Published:** 2020-05-27

**Authors:** Gurdeep S Mannu, Zhe Wang, John Broggio, Jackie Charman, Shan Cheung, Olive Kearins, David Dodwell, Sarah C Darby

**Affiliations:** 1 Nuffield Department of Population Health, University of Oxford, Richard Doll Building, Old Road Campus, Oxford OX3 7LF, UK; 2 National Cancer Registration and Analysis Service, Public Health England, Birmingham, UK

## Abstract

**Objective:**

To evaluate the long term risks of invasive breast cancer and death from breast cancer after ductal carcinoma in situ (DCIS) diagnosed through breast screening.

**Design:**

Population based observational cohort study.

**Setting:**

Data from the NHS Breast Screening Programme and the National Cancer Registration and Analysis Service.

**Participants:**

All 35 024 women in England diagnosed as having DCIS by the NHS Breast Screening Programme from its start in 1988 until March 2014.

**Main outcome measures:**

Incident invasive breast cancer and death from breast cancer.

**Results:**

By December 2014, 13 606 women had been followed for up to five years, 10 998 for five to nine years, 6861 for 10-14 years, 2620 for 15-19 years, and 939 for at least 20 years. Among these women, 2076 developed invasive breast cancer, corresponding to an incidence rate of 8.82 (95% confidence interval 8.45 to 9.21) per 1000 women per year and more than double that expected from national cancer incidence rates (ratio of observed rate to expected rate 2.52, 95% confidence interval 2.41 to 2.63). The increase started in the second year after diagnosis of DCIS and continued until the end of follow-up. In the same group of women, 310 died from breast cancer, corresponding to a death rate of 1.26 (1.13 to 1.41) per 1000 women per year and 70% higher than that expected from national breast cancer mortality rates (observed:expected ratio 1.70, 1.52 to 1.90). During the first five years after diagnosis of DCIS, the breast cancer death rate was similar to that expected from national mortality rates (observed:expected ratio 0.87, 0.69 to 1.10), but it then increased, with values of 1.98 (1.65 to 2.37), 2.99 (2.41 to 3.70), and 2.77 (2.01 to 3.80) in years five to nine, 10-14, and 15 or more after DCIS diagnosis. Among 29 044 women with unilateral DCIS undergoing surgery, those who had more intensive treatment (mastectomy, radiotherapy for women who had breast conserving surgery, and endocrine treatment in oestrogen receptor positive disease) and those with larger final surgical margins had lower rates of invasive breast cancer.

**Conclusions:**

To date, women with DCIS detected by screening have, on average, experienced higher long term risks of invasive breast cancer and death from breast cancer than women in the general population during a period of at least two decades after their diagnosis. More intensive treatment and larger final surgical margins were associated with lower risks of invasive breast cancer.

## Introduction

The incidence of ductal carcinoma in situ (DCIS) has been increasing, particularly in areas where breast screening programmes have been introduced, and DCIS now accounts for approximately one fifth of all new diagnoses of breast cancer detected through screening.^1^ ^2^ ^3^ ^4^ Most women with DCIS are treated with surgery, often followed by radiotherapy and sometimes with endocrine treatment. The long term risks of invasive breast cancer and of death from breast cancer after DCIS detected by screening are incompletely understood. How these risks evolve over time is also unclear, so that the optimal period of post-treatment follow-up and frequency of surveillance imaging both remain uncertain. Additionally, it has been suggested that DCIS is being over-diagnosed and over-treated. These concerns have raised interest in non-operative management of DCIS,^5^ ^6^ ^7^ which requires reliable identification of women at low risk of developing invasive breast cancer and has created a need for information on how the incidence of invasive breast cancer after DCIS varies with the characteristics of the patient, the tumour, and the treatment received.

Several cohort studies of women with DCIS have been conducted, but these lack information on screening status, do not distinguish between recurrence of DCIS and occurrence of invasive breast cancer, have short duration of follow-up, or have only limited information on resection margins, pathological factors, or use of endocrine treatment.^8^ ^9^ ^10^ ^11^ ^12^ Randomised trials of radiotherapy and endocrine treatment have reported the effects of these treatments in women with DCIS,^13^ ^14^ ^15^ but for some aspects of care, such as the determination of optimal margin distance, randomised trials are not feasible. Therefore, to provide further information on the long term consequences of DCIS, we did a population based study characterising the risks of invasive breast cancer and death from breast cancer among all women diagnosed as having DCIS detected by screening in England. We also investigated patient, tumour, and treatment related factors associated with these endpoints.

## Methods

### Study population and data

The National Health Service Breast Screening Programme (NHSBSP),^16^ the first of its kind in the world, began in 1988 and achieved national coverage in 1993. It is a centralised service in which women in specified age groups are sent personal letters every three years inviting them to attend an appointment for screening mammography in one of 78 breast screening units across England. Initially, it used single view mammography and women aged 50-64 years were invited. However, the use of two view mammography was introduced from the mid-1990s, and from 2003 the age range was extended to include women aged 65-70 years. The attendance rate for women invited for screening has consistently been over 70%.

Since the introduction of the NHSBSP, all women diagnosed as having DCIS detected by screening in England have been registered prospectively by organisations that are now unified into the National Cancer Registration and Analysis Service (NCRAS).^17^ On an ongoing basis, NCRAS routinely links these registrations at the patient level with other information on the same woman, including registrations of other cancers, the date of emigration, and the date and cause of death when the woman dies. For this study, the NHSBSP also passed on information on the tumour characteristics and treatments recorded in its annual audits from April 2000 to March 2014 to NCRAS for linkage. The resulting dataset, which included information up to December 2014 on 36 878 women, was then de-personalised and released to the investigators in Oxford for analysis.

The dataset received in Oxford included, for each woman in the study, information on patient related factors (date of screening, date of DCIS diagnosis, age at DCIS diagnosis, any previous cancer diagnoses, region of residence based on the former Cancer Registry regions) and the date and site of any subsequent breast cancer registrations, as well as the date of emigration and the date and cause of death if relevant. For women screened from April 2000, information was also received on tumour related factors (DCIS tumour size, grade, laterality, oestrogen receptor status) and treatments (type of surgery, radiotherapy, endocrine treatment). Margin assessment was carried out in the pathology departments of individual treating centres/hospitals under national guidelines that have been in place since at least 2002, and information on final surgical margin status was available for women diagnosed as having DCIS from 2007 onwards. For women who had more than one operation, the final margin distance was calculated as the sum of the final closest margin distances recorded for each operation. To provide reassurance that women included in the study had, in fact, been initially diagnosed as having DCIS rather than invasive breast cancer, two clinicians (GSM and DD) examined the textual pathology reports stored by NCRAS for a sample of 130 women for whom invasive breast cancer or death from breast cancer was subsequently reported; none was found in which the initial diagnosis had in fact been invasive breast cancer rather than DCIS.

### Analysis

**Fig 1:**
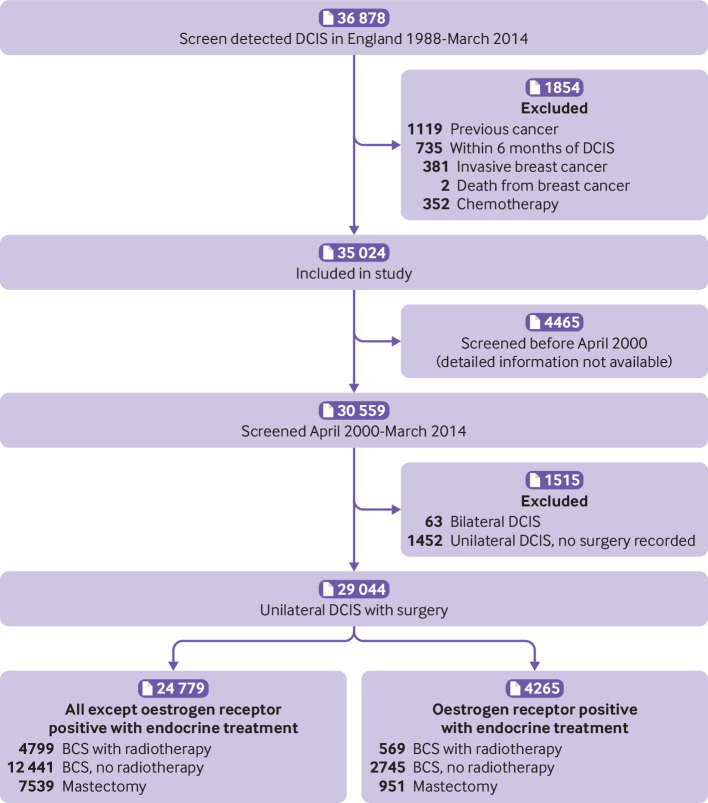
Derivation of study population. BCS=breast conserving surgery; DCIS=ductal carcinoma in situ

We excluded any woman recorded as having an invasive cancer (other than non-melanoma skin cancer) before her diagnosis of DCIS, as well as women registered with invasive breast cancer or death from breast cancer or recorded as receiving chemotherapy within six months of diagnosis of DCIS (fig 1). We calculated cumulative observed risks of invasive breast cancer and death from breast cancer and cumulative rates of invasive breast cancer by considering women from six months after their DCIS diagnosis until the earliest of diagnosis of invasive breast cancer or death, loss to follow-up, or 31 December 2014.^18^ We calculated cumulative expected risks similarly, using cancer incidence rates for England and mortality rates for England and Wales in five year age groups and single calendar years. We took into account competing risks of death from other causes by using 2014 death rates for England and Wales. Confidence intervals for cumulative risks, observed and cumulative rates, and ratios of observed to expected rates were based on the Poisson distribution.^19^

We also used Poisson regression for analyses requiring adjustments and tests for interactions. However, information was missing for some women for the variables tumour size, DCIS grade, oestrogen receptor status, and tumour laterality. Omitting these women from the analysis may lead to loss of precision and possible bias. Therefore, we did analyses including these variables in two different ways. Firstly, we assigned the missing values for each variable to a separate category. Secondly, we did analyses using multiple imputation for the missing values. This takes account of any correlations between the missing variable and variables that are known. It also allows for the uncertainty arising from the missing variables in standard errors and significance tests. Results from the two different methods were virtually identical, and those presented in the paper are based on multiple imputation. We used Stata statistical software version 15.1 and R version 3.2.2 for analyses. Further details of the statistical methods are provided in supplementary text S1.

### Patient and public involvement

This study comprises a statistical analysis conducted on routinely collected data that had been depersonalised. No patients were involved in setting the research question or the outcome measures, nor were they involved in developing plans for the study. The results have already been presented to representatives of appropriate patient groups. After publication, they will be freely available both to stakeholders and to the broader public.

## Results

### Characteristics of study population and mortality from all causes

By March 2014, a total of 35 024 women in England had been diagnosed as having DCIS detected by screening as their first cancer and were included in the study (fig 1). Thirty two per cent were aged below 55 years at diagnosis, 22% were aged 55-59, 23% were 60-64, and 24% were at least 65 years (table 1). By December 2014, 13 606 women had been followed for less than five years, 10 998 for five to nine years, 6861 for 10-14 years, 2620 for 15-19 years, and 939 for 20 years or more. A total of 2234 women had died and, as would be expected in a population attending for screening, their overall mortality was lower than that expected from mortality rates in the general population (ratio of observed rate to expected rate based on five year age groups in single calendar years (observed:expected ratio) 0.77, 95% confidence interval 0.74 to 0.81; P<0.001) (tables S3 and S4).

**Table 1:** Characteristics of 35 024 women in England diagnosed as having ductal carcinoma in situ (DCIS) as a result of screening between January 1988 and March 2014, and their status on 31 December 2014. Values are numbers (percentages)

	Screened before April 2000 (n=4465)	Screened April 2000 to March 2014					Total (n=35 024)	
		Bilateral DCIS (n=63)	Unilateral DCIS					
			BCS+RT (n=5368)	BCS-RT (n=15 188)	Mastectomy (n=8488)	No surgery recorded (n=1452)		
**Year of screening **							
Before April 2000	4465 (100.0)	—	—	—	—	—	4465 (12.7)
April 2000-Dec 2004	—	14 (22)	669 (12.5)	3617 (23.8)	1985 (23.4)	557 (38.4)	6842 (19.5)
Jan 2005-Dec 2009	—	23 (37)	1313 (24.5)	5908 (38.9)	3203 (37.7)	320 (22.0)	10 767 (30.7)
Jan 2010-March 2014	—	26 (41)	3386 (63.1)	5663 (37.3)	3300 (38.9)	575 (39.6)	12 950 (37.0)
**Age at DCIS diagnosis, years**							
<55	1630 (36.5)	19 (30)	1611 (30.0)	4577 (30.1)	2829 (33.3)	459 (31.6)	11 125 (31.8)
55-59	1234 (27.6)	16 (25)	1122 (20.9)	3107 (20.5)	1820 (21.4)	304 (20.9)	7603 (21.7)
60-64	1234 (27.6)	10 (16)	1183 (22.0)	3446 (22.7)	1789 (21.1)	329 (22.7)	7991 (22.8)
≥65	367 (8.2)	18 (29)	1452 (27.0)	4058 (26.7)	2050 (24.2)	360 (24.8)	8305 (23.7)
**Region**							
Eastern	—	6 (10)	1367 (25.5)	1315 (8.7)	978 (11.5)	118 (8.1)	3784 (10.8)
North West	—	5 (8)	456 (8.5)	2117 (13.9)	1105 (13.0)	119 (8.2)	3802 (10.9)
Northern/Yorkshire	—	9 (14)	1758 (32.7)	1176 (7.7)	1433 (16.9)	88 (6.1)	4464 (12.7)
Oxford	—	5 (8)	161 (3.0)	1052 (6.9)	450 (5.3)	38 (2.6)	1706 (4.9)
South West	—	10 (16)	216 (4.0)	3162 (20.8)	1327 (15.6)	108 (7.4)	4823 (13.8)
Thames	—	15 (24)	607 (11.3)	3472 (22.9)	1600 (18.9)	396 (27.3)	6090 (17.4)
Trent	—	5 (8)	413 (7.7)	1242 (8.2)	824 (9.7)	532 (36.6)	3016 (8.6)
West Midlands	—	8 (13)	390 (7.3)	1652 (10.9)	771 (9.1)	53 (3.7)	2874 (8.2)
Unknown	4465 (100.0)	—	—	—	—	—	4465 (12.7)
**Tumour size, mm**							
≤10	—	14 (22)	1595 (29.7)	7085 (46.6)	853 (10.0)	390 (26.9)	9937 (28.4)
11-20	—	14 (22)	2147 (40.0)	4820 (31.7)	1687 (19.9)	352 (24.2)	9020 (25.8)
21-50	—	18 (29)	1551 (28.9)	3056 (20.1)	4034 (47.5)	309 (21.3)	8968 (25.6)
≥51	—	9 (14)	75 (1.4)	227 (1.5)	1914 (22.5)	56 (3.9)	2281 (6.5)
Unknown	4465 (100.0)	8 (13)	—	—	—	345 (23.8)	4818 (13.8)
**DCIS grade**							
Low/intermediate	—	23 (37)	1375 (25.6)	8019 (52.8)	2337 (27.5)	535 (36.8)	12 289 (35.1)
High	—	33 (52)	3993 (74.4)	7169 (47.2)	6151 (72.5)	680 (46.8)	18 026 (51.5)
Unknown	4465 (100.0)	7 (11)	—	—	—	237 (16.3)	4709 (13.4)
**Oestrogen receptor status and endocrine treatment **							
ER+, no endocrine	—	21 (33)	3585 (66.8)	9947 (65.5)	5003 (58.9)	277 (19.1)	18 833 (53.8)
ER+, endocrine	—	10 (16)	569 (10.6)	2747 (18.1)	950 (11.2)	245 (16.9)	4521 (12.9)
ER-	—	7 (11)	1214 (22.6)	2494 (16.4)	2535 (29.9)	127 (8.7)	6377 (18.2)
Unknown	4465 (100.0)	25 (40)	—	—	—	803 (55.3)	5293 (15.1)
**Laterality of DCIS**							
Left	2230 (49.9)	—	2802 (52.2)	7821 (51.5)	4365 (51.4)	748 (51.5)	17 966 (51.3)
Right	2049 (45.9)	—	2566 (47.8)	7367 (48.5)	4123 (48.6)	691 (47.6)	16 796 (48.0)
Bilateral	13 (0.3)	63 (100)	—	—	—	—	76 (0.2)
Unknown	173 (3.9)	—	—	—	—	13 (0.9)	186 (0.5)
**Length of follow-up, years**							
0-4	162 (3.6)	27 (43)	3428 (63.9)	5939 (39.1)	3441 (40.5)	609 (41.9)	13 606 (38.8)
5-9	219 (4.9)	23 (37)	1316 (24.5)	5898 (38.8)	3214 (37.9)	328 (22.6)	10 998 (31.4)
10-14	525 (11.8)	13 (21)	624 (11.6)	3351 (22.1)	1833 (21.6)	515 (35.5)	6861 (19.6)
15-19	2620 (58.7)	—	—	—	—	—	2620 (7.5)
≥20	939 (21.0)	—	—	—	—	—	939 (2.7)
**Invasive breast cancer diagnosed by 31 Dec 2014**							
Ipsilateral	320 (7.2)	—	73 (1.4)	460 (3.0)	113 (1.3)	63 (4.3)	1029 (2.9)
Contralateral	234 (5.2)	—	84 (1.6)	297 (2.0)	206 (2.4)	39 (2.7)	860 (2.5)
Laterality unknown	89 (2.0)	5 (8)	7 (0.1)	41 (0.3)	35 (0.4)	10 (0.7)	187 (0.5)
**Vital status on 31 Dec 2014**							
Alive	3363 (75.3)	60 (95)	5220 (97.2)	14 472 (95.3)	8103 (95.5)	1345 (92.6)	32 563 (93.0)
Emigrated	151 (3.4)	—	6 (0.1)	46 (0.3)	22 (0.3)	2 (0.1)	227 (0.6)
Dead	951 (21.3)	3 (5)	142 (2.6)	670 (4.4)	363 (4.3)	105 (7.2)	2234 (6.4)
**Cause of death**							
Breast cancer	150	1	16	67	53	23	310
Other known causes	725	2	126	595	300	79	1827
Unknown cause	76	0	0	8	10	3	97

BCS+RT=breast conserving surgery, radiotherapy recorded; BCS-RT=breast conserving surgery, radiotherapy not recorded; ER+, no endocrine=oestrogen receptor positive DCIS, endocrine therapy not recorded; ER+, endocrine=oestrogen receptor positive DCIS, endocrine therapy recorded; ER-=oestrogen receptor negative DCIS.

### Incidence of invasive breast cancer

By 31 December 2014, 2076 women in the study had developed invasive breast cancer (1029 ipsilateral, 860 contralateral, 187 laterality unknown) (table 1). The rate of invasive breast cancer was 8.82 (95% confidence interval 8.45 to 9.21) per 1000 women per year and did not vary significantly with age at diagnosis of DCIS (P for trend=0.52) (table S5). The rate of invasive breast cancer was, however, more than double that expected from breast cancer incidence rates in the general population (observed:expected ratio 2.52, 2.41 to 2.63; P<0.001). The observed to expected ratio was 1.06 (0.81 to 1.37) in the period 0.5-0.9 years after DCIS diagnosis, increasing to 2.13 (1.86 to 2.44) during years 1.0-1.9 and to 2.67 (2.35 to 3.02) during years 2.0-2.9. Beyond three years, it was 2.69 (2.56 to 2.83) with no significant trend with time (P for trend=0.53). After accounting for age at DCIS diagnosis and time since diagnosis, we observed no significant trend in the observed to expected ratio with calendar year of DCIS diagnosis (P for trend=0.26) (table S6).

**Fig 2:**
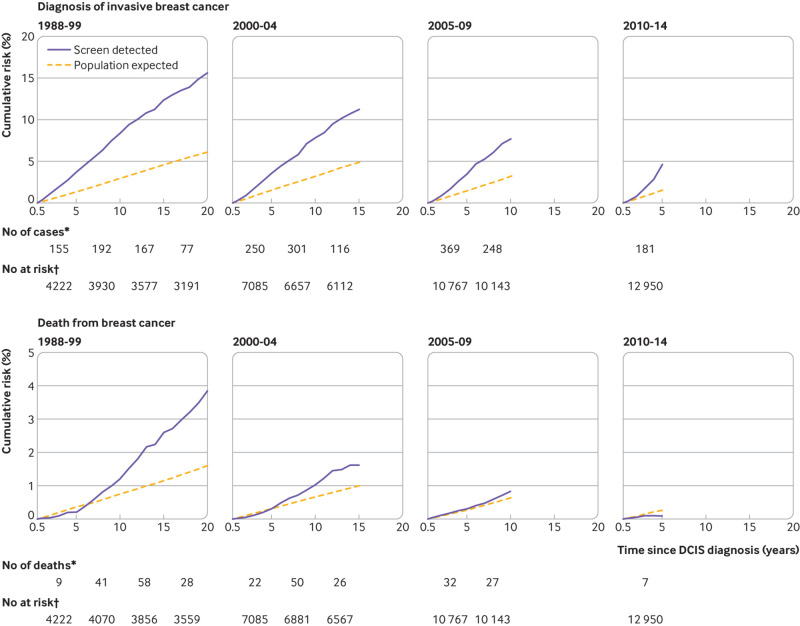
Cumulative risk diagnosis of invasive breast cancer (top) and of death from breast cancer (bottom) in 35 024 women with ductal carcinoma in situ (DCIS) detected through screening by year of diagnosis of DCIS. Cumulative risks take into account competing risks from other causes of death. Expected values are based on cancer incidence rates for England and mortality rates for England and Wales. See supplementary text S1 and supplementary tables S7 and S11 for further details. BCS=breast conserving surgery; DCIS=ductal carcinoma in situ. *Number of invasive breast cancers/deaths from breast cancer during interval. †Number of women at risk of invasive breast cancer/death from breast cancer at start of interval

By the end of the third year after diagnosis, the cumulative risk of invasive breast cancer exceeded that expected in every calendar period studied, and observed and expected cumulative risks continued to diverge with increasing time since diagnosis of DCIS (fig 2 and table S7). By 10 years after diagnosis of DCIS, the cumulative risks of invasive breast cancer were 8.3% (95% confidence interval 7.5% to 9.2%), 7.8% (7.2% to 8.4%), and 7.7% (6.9% to 8.4%), compared with 2.9%, 3.2%, and 3.2% expected for women with diagnosis of DCIS before the year 2000 and during years 2000-04 and 2005-09, respectively. By 20 years after diagnosis of DCIS, the cumulative risk of invasive breast cancer was 15.6% (14.3% to 16.8%) compared with 6.1% expected for women with diagnosis before 2000.

Among the 30 559 women screened during April 2000 to March 2014, the observed to expected ratio for invasive breast cancer was 2.43 (2.31 to 2.56) overall and 2.68 (2.52 to 2.85) in the period three or more years after diagnosis of DCIS (table S8). In the period three or more years after diagnosis of DCIS, the observed to expected ratio was 2.63 (2.47 to 2.81) for women with unilateral DCIS who received surgery; for women with unilateral DCIS and no surgery recorded it was 3.38 (2.70 to 4.24), and for women with bilateral DCIS it was 3.96 (1.28 to 12.30) (P for heterogeneity=0.11).

### Mortality from breast cancer

By 31 December 2014, 310 women had died with breast cancer as the certified cause of death (table 1). The rate of death from breast cancer was 1.26 (1.13 to 1.41) per 1000 women per year and did not vary significantly with age at diagnosis of DCIS (P for trend=0.20) (table S9). The rate was, however, greater than expected from national breast cancer mortality rates (observed:expected ratio 1.70, 1.52 to 1.90; P<0.001). The observed to expected ratio during the first five years after diagnosis of DCIS was close to one (0.87, 0.69 to 1.10), but during years five to nine it was nearly doubled (1.98, 1.65 to 2.37), and it was higher still during years 10-14 (2.99, 2.41 to 3.70) and 15 or more (2.77, 2.01 to 3.80). After accounting for age at diagnosis and time since diagnosis, we found weak evidence that the observed to expected ratio had decreased with increasing calendar year of diagnosis (P for trend=0.09) (table S10).

During the first five years after diagnosis of DCIS, the cumulative risk of death from breast cancer was similar to that expected, but after that the cumulative observed risk increased more rapidly than expected (fig 2, table S11). At 10 years after diagnosis of DCIS, the cumulative risks of death from breast cancer were 1.2% (0.9% to 1.5%), 1.0% (0.8% to 1.2%), and 0.8% (0.5% to 1.1%), compared with 0.7%, 0.7%, and 0.6% expected for women diagnosed before 2000, during 2000-04, and during 2005-09, respectively. By 20 years after diagnosis of DCIS, the cumulative risk of death from breast cancer was 3.8% (3.2% to 4.5%) compared with 1.6% expected for women with diagnosis before 2000.

Among the 30 559 women screened during April 2000 to March 2014, the observed to expected ratio for death from breast cancer was 1.36 (1.16 to 1.58) overall and 1.88 (1.54 to 2.29) in the period five or more years after diagnosis of DCIS (table S12). In the five years or more period, the observed to expected ratio was 1.72 (1.38 to 2.12) for women with unilateral DCIS who had surgery; for women with unilateral DCIS and no surgery recorded it was higher, at 3.89 (2.31 to 6.57), and for women with bilateral DCIS it was higher still, at 9.96 (1.40 to 70.7) (P for heterogeneity=0.02).

### Invasive breast cancer after unilateral DCIS and surgery but without endocrine treatment

A total of 24 779 women were diagnosed as having DCIS detected by screening during April 2000 to March 2014 and recorded as undergoing surgery but not receiving endocrine treatment (fig 1). Among these women, 4799 were recorded as having breast conserving surgery with radiotherapy, 12 441 as having breast conserving surgery with no record of radiotherapy, and 7539 as having mastectomy. The characteristics of the women in the three treatment groups differed significantly for every recorded characteristic (P for heterogeneity<0.001), with the exception of DCIS laterality (P for heterogeneity=0.36) (table S13). By 31 December 2014, 564 of these 24 779 women had been diagnosed as having ipsilateral invasive breast cancer.

**Fig 3:**
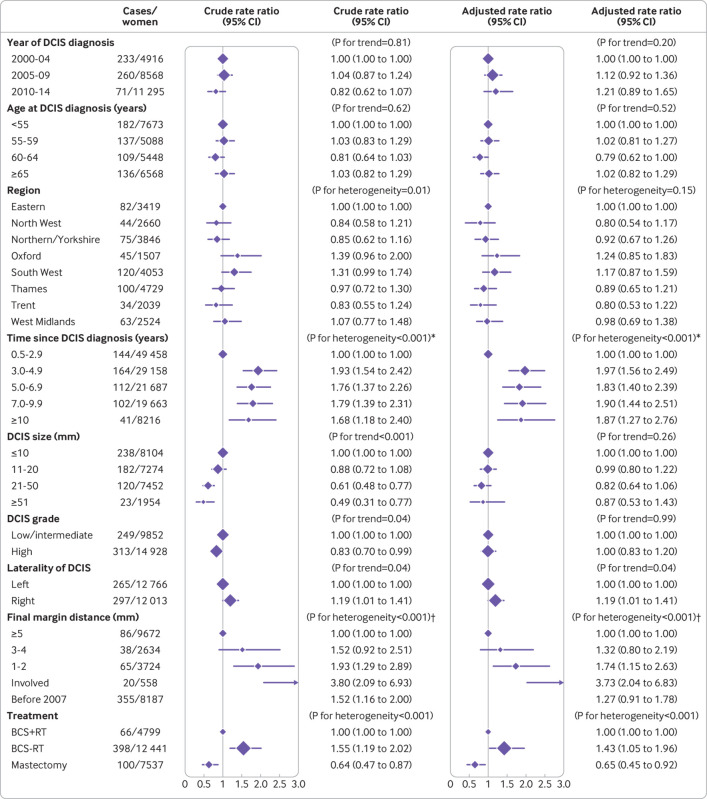
Incidence of ipsilateral invasive breast cancer according to various factors in 24 779 women diagnosed as having unilateral ductal carcinoma in situ (DCIS) as a result of screening during April 2000 to March 2014 and who had surgery. Women with oestrogen receptor positive DCIS and recorded as receiving endocrine treatment were excluded. For each factor, rates are shown relative to the first category shown and adjustment is for all other factors except final margin distance. Final margin distance was not included in adjustment as information on this variable was available only from 2007 onwards. Separate results for “breast conserving surgery with radiotherapy,” for “breast conserving surgery, radiotherapy not recorded,” and for “mastectomy” and results showing final margin distances of 1 mm and 2 mm separately are given in supplementary figures S1 to S3. BCS+RT=breast conserving surgery, radiotherapy recorded; BCS-RT: breast conserving surgery, radiotherapy not recorded. *Tests for trend excluding years 0.5-2.9: crude P=0.40; adjusted P=0.87. †Tests for trend across clear margin categories: crude P=0.001; adjusted P=0.009

**Fig 4:**
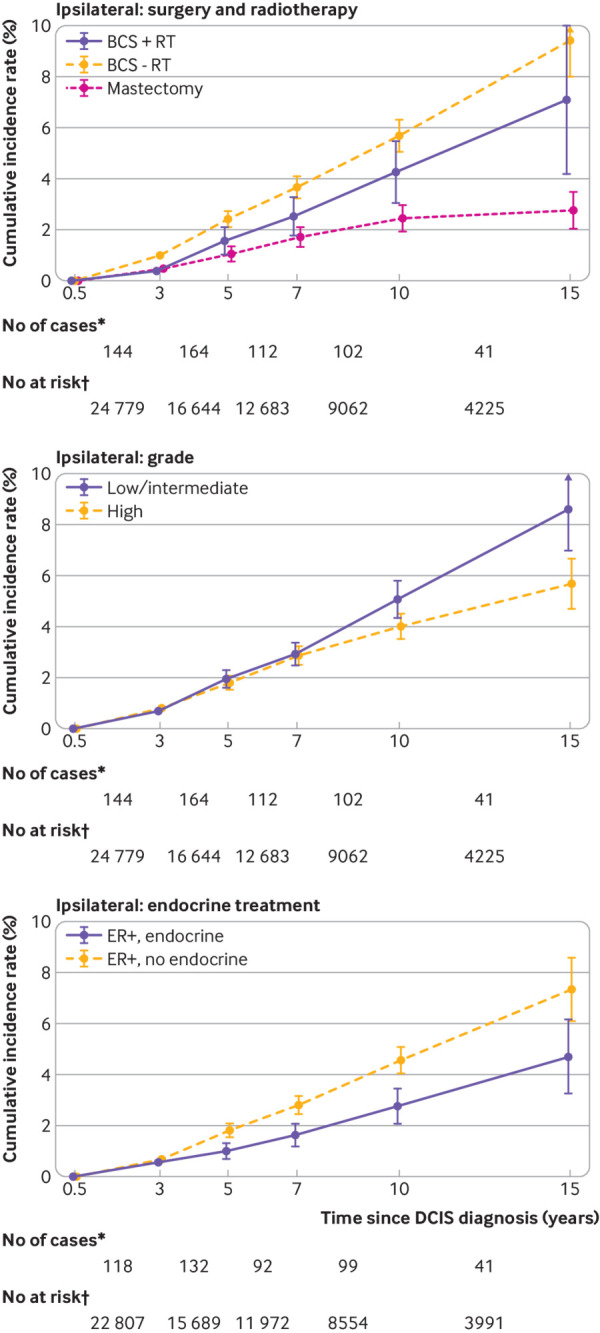
Cumulative incidence rates of ipsilateral invasive breast cancer and 95% confidence intervals in 29 044 women with unilateral ductal carcinoma in situ (DCIS) detected as a result of screening during April 2000 to March 2014 and who had surgery. Women with oestrogen receptor positive DCIS and recorded as receiving endocrine treatment were excluded from the top two graphs. BCS+RT=breast conserving surgery, radiotherapy recorded; BCS-RT=breast conserving surgery, radiotherapy not recorded; ER+, endocrine=oestrogen receptor positive DCIS and endocrine treatment recorded; ER+, no endocrine=oestrogen receptor positive DCIS and endocrine treatment not recorded. *Number of ipsilateral invasive breast cancers during interval; †Number of women at risk of ipsilateral invasive breast cancer at start of interval

Compared with women having breast conserving surgery with radiotherapy, the rate of ipsilateral invasive breast cancer for women having breast conserving surgery with no record of radiotherapy was higher (adjusted rate ratio 1.43, 95% confidence interval 1.05 to 1.96), whereas for women having mastectomy it was lower (0.65, 0.45 to 0.92) (P for heterogeneity<0.001; fig 3). The rate of ipsilateral invasive breast cancer was lower during the first three years after diagnosis of DCIS than subsequently (fig 3). After three years, however, the cumulative rate of invasive breast cancer increased more steeply for women having breast conserving surgery (with or without radiotherapy) than for those having mastectomy (fig 4).

The rate of ipsilateral invasive breast cancer was strongly associated with final margin status. Compared with women whose final margin distance was at least 5 mm, the adjusted rate ratio for women with involved margins was 3.73 (2.04 to 6.83), whereas for women with final margin distance 1-2 mm it was 1.74 (1.15 to 2.63) and for those with final margin distance 3-4 mm it was 1.32 (0.80 to 2.19) (P for trend=0.009 across clear margin categories) (fig 3). When we repeated the analysis considering final margin distances of 1 mm and 2 mm separately, the adjusted rate ratios for margins of 1 mm and 2 mm compared with 5 mm or more were 1.47 (0.85 to 2.54) and 2.02 (1.23 to 3.31), respectively (figure S1). We found similar associations with final margin distance when we examined women having breast conserving surgery with radiotherapy, breast conserving surgery with no record of radiotherapy, and mastectomy separately, but only for breast conserving surgery with no record of radiotherapy were the numbers sufficient for the trend across clear margin categories to reach statistical significance (P for trend=0.05 across clear margin categories) (figure S2).

Women with low/intermediate grade DCIS were less likely than women with high grade DCIS to have mastectomy (20.3% *v* 37.1%; table S13). Although we observed no overall difference in the adjusted rate of ipsilateral invasive breast cancer between women with low/intermediate DCIS and women with high grade DCIS (fig 3), a significant interaction existed between grade and time since diagnosis (P=0.01) (table S14). In the period more than seven years after diagnosis of DCIS, the cumulative rate of ipsilateral invasive breast cancer increased more rapidly in women with low/intermediate grade DCIS than in women with high grade DCIS (fig 4). This was due to higher rates of invasive breast cancer in women with low/intermediate grade DCIS who had had breast conserving surgery rather than mastectomy, irrespective of whether they also had radiotherapy (figure S4).

The rate of ipsilateral invasive breast cancer was slightly higher for women with more recent diagnosis (adjusted rate ratio 1.21, 0.89 to 1.65, for 2010-14 *v* 2000-04), but the trend across calendar periods was not significant (P for trend=0.20) (fig 3). We observed no significant overall trend in the rate of ipsilateral invasive breast cancer with age at diagnosis of DCIS or tumour size, and after adjustment no significant heterogeneity existed according to region of residence, although we found some evidence of an association between rate of ipsilateral invasive breast cancer and laterality of DCIS (P for trend=0.04). Additional results are shown in table S14 and figure S5.

**Fig 5:**
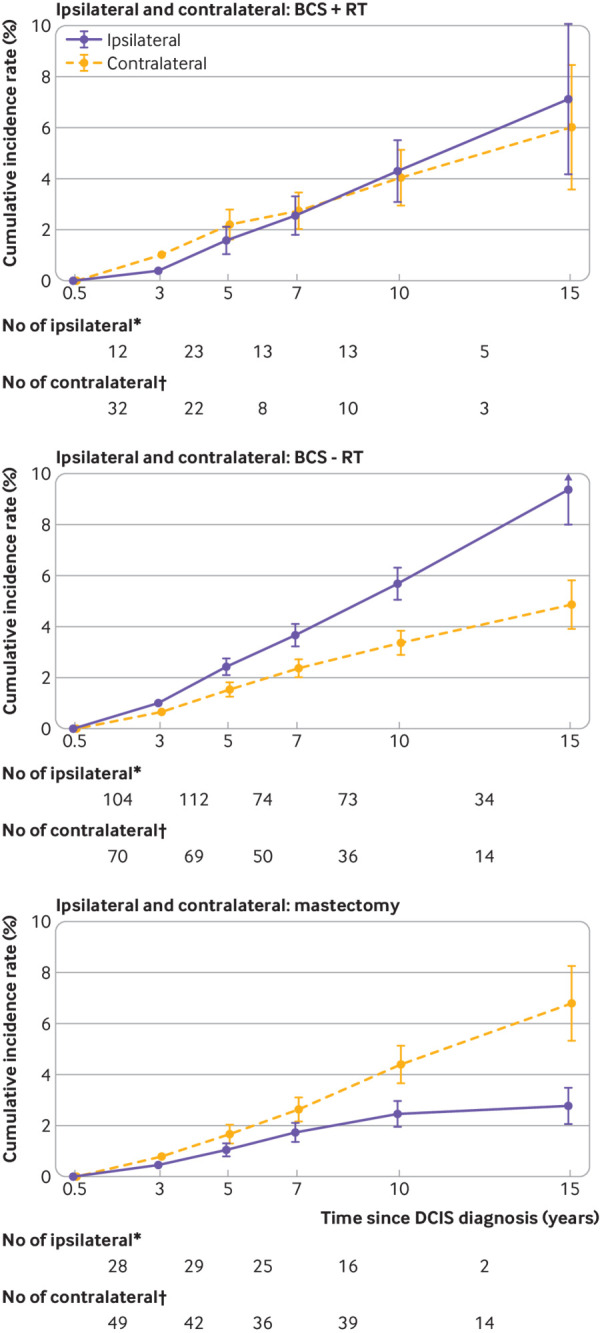
Cumulative incidence rates of ipsilateral and contralateral invasive breast cancer and 95% confidence intervals in 24 716 women with unilateral ductal carcinoma in situ (DCIS) detected as a result of screening between April 2000 and March 2014 and who had surgery. Women with unknown laterality of subsequent invasive cancer were excluded, as were women with oestrogen receptor positive DCIS and recorded as receiving endocrine treatment. BCS+RT=breast conserving surgery, radiotherapy recorded; BCS-RT=breast conserving surgery, radiotherapy not recorded. *Number of ipsilateral invasive breast cancers during interval. †Number of contralateral invasive breast cancers during interval

As with ipsilateral invasive breast cancer, the incidence rate of contralateral invasive breast cancer was lower during the first three years after diagnosis of DCIS than subsequently, but after this we observed no significant trend (P for trend=0.36) and only weak evidence that it varied with treatment (P for heterogeneity=0.04) (fig S6). In contrast, we found strong evidence that the ratio of the ipsilateral to the contralateral invasive breast cancer rate varied with treatment (P for heterogeneity<0.001) (fig S7). For women having breast conserving surgery with radiotherapy, the cumulative incidence rates of ipsilateral and contralateral invasive breast cancer by 15 years after diagnosis of DCIS were similar (ipsilateral: 7.1%, 4.2% to 10.0%; contralateral: 6.0%, 3.5% to 8.5%), whereas for women having breast conserving surgery with no record of radiotherapy the 15 year cumulative incidence rate of ipsilateral invasive breast cancer was higher than that for contralateral invasive breast cancer (ipsilateral: 9.4%, 8.0% to 10.8%; contralateral: 4.9%, 3.9% to 5.8%), and for women having mastectomy it was lower (ipsilateral: 2.8%, 2.1% to 3.5%; contralateral: 6.8%, 5.3% to 8.3%) (fig 5).

### Invasive breast cancer after unilateral DCIS and surgery with endocrine treatment

Of the 29 044 women screened during April 2000 to March 2014 and recorded as having unilateral DCIS and undergoing surgery, 18 542 had oestrogen receptor positive disease but did not receive endocrine treatment, 4265 had oestrogen receptor positive disease and received endocrine treatment, and 6237 women had oestrogen receptor negative disease (table S15). The rate of ipsilateral invasive breast cancer varied between these three groups (P for heterogeneity<0.001) (fig S8). Compared with women with oestrogen receptor positive disease who did not receive endocrine treatment, the rate for women with oestrogen receptor negative disease was similar (adjusted rate ratio 1.16, 0.90 to 1.50), but the rate for women with oestrogen receptor positive disease who received endocrine treatment was lower (adjusted rate ratio 0.62, 0.49 to 0.80). By 15 years after diagnosis of DCIS, the cumulative rate of ipsilateral invasive breast cancer was 4.7% (3.2% to 6.2%) in women with oestrogen receptor positive disease who received endocrine treatment compared with 7.3% (6.1% to 8.5%) in women who did not receive it (fig 4). Among women with oestrogen receptor positive disease who were treated with breast conserving surgery with radiotherapy, the invasive breast cancer rate was lower for those who also received endocrine treatment than for those who did not, but the numbers were small so the difference was not statistically significant (adjusted rate ratio 0.61, 0.35 to 1.08) (figure S9).

### Breast cancer mortality after unilateral DCIS and surgery

**Fig 6:**
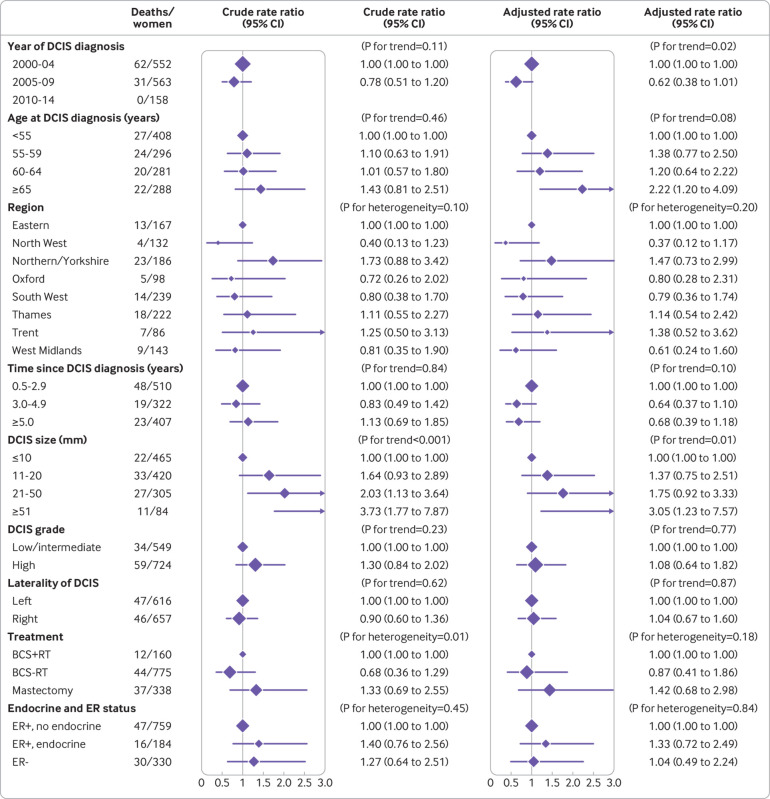
Breast cancer mortality among 1273 women registered with invasive breast cancer after previously being diagnosed as having unilateral ductal carcinoma in situ (DCIS) as a result of screening during April 2000 to March 2014 and who had surgery, according to characteristics of original DCIS. For each variable, adjustment was for all other variables shown. BCS+RT=breast conserving surgery, radiotherapy recorded; BCS-RT=breast conserving surgery, radiotherapy not recorded; ER+, endocrine=oestrogen receptor positive DCIS and endocrine treatment recorded; ER+, no endocrine=oestrogen receptor positive DCIS and endocrine treatment not recorded; ER-=oestrogen receptor negative

Of the 29 044 women screened during April 2000 to March 2014, diagnosed as having unilateral DCIS, and recorded as undergoing surgery, 1316 had been registered with invasive breast cancer by 31 December 2014, of which 43 were death certificate only registrations. Among the remaining 1273 women, 93 died with breast cancer as the certified cause of death. Women whose DCIS was diagnosed more recently had a lower breast cancer mortality rate than those diagnosed earlier (P for trend=0.02), and women whose DCIS was larger had a higher breast cancer mortality rate than women whose DCIS was smaller (P for trend=0.01) (fig 6). No other characteristics of the original DCIS were significantly associated with the breast cancer mortality rate.

## Discussion

We have shown that women diagnosed as having DCIS detected by screening in England have experienced substantially increased risks of both invasive breast cancer and death from breast cancer compared with women in the general population, despite having lower overall mortality. The women with DCIS detected by screening had rates of both invasive breast cancer and death from breast cancer that, from a few years after diagnosis of DCIS, were more than double those of the general population, and the increases lasted until at least 20 years after diagnosis. The increases affected women of all ages and applied to women with unilateral DCIS who were treated surgically, as well as to the few women with bilateral DCIS or for whom no surgery was reported. So far, little evidence is available to suggest that rates of invasive breast cancer are lower for women whose DCIS was diagnosed more recently, but rates of death from breast cancer for women with more recent diagnosis are decreasing, probably reflecting improved treatment of invasive disease.

We did not find any significant differences in mortality from breast cancer between the various treatment groups. However, women with unilateral DCIS undergoing breast conserving surgery had higher rates of ipsilateral invasive breast cancer than did those undergoing mastectomy, and the absolute difference in the cumulative incidence rate between these two groups continued to increase for at least 15 years after the diagnosis of DCIS. Among women having breast conserving surgery, rates of ipsilateral invasive breast cancer were lower for those who also had radiotherapy, but even with radiotherapy the 15 year cumulative incidence rate was substantially higher than that among women having mastectomy, and only for women recorded as having mastectomy was the 15 year cumulative rate of ipsilateral invasive breast cancer lower than that for contralateral invasive breast cancer. In oestrogen receptor positive disease, rates of invasive breast cancer were considerably lower among women who received endocrine treatment, mainly owing to a reduction in ipsilateral events.

Our study also shows the importance of sufficient margin width. Involved margins were associated with the highest rate of invasive breast cancer, and the rate fell steadily with increasing margin width up to at least 5 mm among women with clear margins. Margins were particularly important after breast conserving surgery with no record of radiotherapy, but we saw similar trends after both breast conserving surgery with radiotherapy and mastectomy.

Finally, our study found higher 15 year cumulative rates of ipsilateral invasive breast cancer among women with low/intermediate grade tumours compared with high grade tumours. This may reflect a continuing risk of progression in low/intermediate grade tumours, as has been reported previously,^20^ or it might reflect under-treatment of these women, as most of them received breast conserving surgery with no record of radiotherapy. In either case, it clearly shows the long term malignant potential of low/intermediate grade tumours.

### Strengths and limitations of study

This is the first study to characterise the long term risks of invasive breast cancer and death from breast cancer in all women diagnosed as having DCIS in a large population based screening programme. We have considered only invasive recurrences and not recurrences of DCIS, because survival after invasive recurrence is substantially poorer than after recurrent DCIS.^12^ Also, whereas the mechanisms developed by NCRAS enable virtually complete follow-up for invasive breast cancer, death from breast cancer, and deaths from other causes, uncertainty remains about the identification of recurrences of DCIS in terms of both sensitivity and specificity. As the screening programme has complete coverage of the population, incidence and mortality rates from the general population of England comprise those for all women invited for screening in the relevant age groups, so they form an appropriate comparison group.

In our analyses, we have made strenuous efforts to control for confounding; however, despite this, great care is always needed before attributing causality to associations identified from an observational study. The data available to us also have some limitations. No central records exist of subsequent breast cancer incidence or mortality in individual women who attended for screening but for whom no abnormality was identified, so we could not do any comparisons involving this group. Other limitations in our data were mainly in the form of missing values. Information on treatment and tumour related factors was available only from 2000 and information for final margin status only from 2007. Even after these dates, this information was missing for a few women. However, multiple imputation has enabled us to include all these women in the analysis in an appropriate way. Cause of death was missing for a few women, so that our estimated rates for death from breast cancer may be slightly low. Also, in some cases in which surgery, radiotherapy, or endocrine treatment was not recorded, it may have been given, so that the true differences between women with and without these treatments are likely to be slightly larger than our estimates. Finally, information on type and duration of endocrine treatment and on any adverse events that women may have experienced while taking it were not available. Information on comorbidities, ethnicity, and deprivation was not available in our dataset. Despite these weaknesses, the overall quality of the data in our study is high.

### Comparison with other studies

A previous study of 108 196 women with DCIS in the United States also reported an increased breast cancer mortality rate compared with the general population.^21^ In that study, which could not distinguish between DCIS detected by screening and DCIS detected by other means, the observed to expected ratio based on breast cancer mortality for all women in the US was 1.8 (95% confidence interval 1.7 to 1.9), slightly higher than the value we report here (1.70, 1.52 to 1.90). Lower rates of ipsilateral invasive breast cancer after breast conserving surgery with radiotherapy compared with breast conserving surgery with no record of radiotherapy have been documented in several randomised trials^15^; when taken together with our study, the combined evidence suggests that the reduction in ipsilateral invasive breast cancer from radiotherapy seen in participants selected for enrolment into randomised trials is likely to apply to women diagnosed as having DCIS in the general population. Notably, both this study and a population based cohort of 10 090 women in the Netherlands with DCIS detected by screening or by other means found much lower rates of ipsilateral invasive breast cancer after mastectomy than after breast conserving surgery, with or without radiotherapy.^8^

Of the two randomised trials of endocrine treatment versus no endocrine treatment after DCIS, one reported a benefit in terms of invasive breast cancer.^13^ ^14^ Endocrine treatment was not recommended for any women with DCIS in the UK until recently,^22^ and many of the women receiving endocrine treatment in our study are likely to have done so as part of a randomised trial, so our findings cannot be regarded as reflecting the likely experience of the general population.

Several studies have reported that women with clear margins after surgery for DCIS have lower rates of local recurrence than do women who have involved margins,^12^ ^23^ ^24^ ^25^ as does our study. Although guidelines recommend that margin widths do not need to be more than 1 or 2 mm,^22^ ^26^ our study suggests that having margins greater than this may confer some further benefit. Further studies examining margin distances in DCIS may be helpful provided that they differentiate between occurrences of invasive breast cancer and recurrences of DCIS, consider precise margin width rather than just broad categories, consider different treatment groups separately, and have sufficient power in terms of numbers of women and length of follow-up.

Our finding of higher breast cancer mortality in invasive disease occurring after larger DCIS tumour size has not previously been reported, and confirmation is needed in other studies before we can conclude that the association is causal. We present outcomes after DCIS detected by screening, but a previous smaller study focusing solely on the West Midlands region of England found that the risk of subsequent invasive breast cancer was higher when DCIS was diagnosed outside the NHSBSP.^27^ As the same finding would probably be the case nationwide, the risks of invasive breast cancer and death from breast cancer found in our study are likely to be lower than those in DCIS not detected by screening.

Although our results suggest that treatment generally reduces the risk of invasive breast cancer for women with DCIS, we acknowledge that some groups of women with favourable characteristics may exist for whom treatment may not be necessary. The overall benefits and risks of treatment can be reliably evaluated only in the setting of randomised trials with long term follow-up. Several trials randomising such women to either standard or non-operative management of low risk DCIS are underway.^28^

### Conclusion and policy implications

Surveillance of women after a diagnosis of DCIS focuses just on the first few years. In the UK, for example, most women are recalled for yearly surveillance mammograms for five years, after which further follow-up is three yearly via the national screening programme up to age 70 years.^22^ We have, however, provided evidence of the long term nature of the risk of invasive disease after a diagnosis of DCIS, even for women with low or intermediate grade disease. Our results also show that women who have had a mastectomy have a lower long term risks of invasive disease than do those who had breast conserving surgery, even when accompanied by radiotherapy.

**What is already known on this topic**

The incidence of ductal carcinoma in situ (DCIS) has increased substantially in recent years, especially since the introduction of breast screening programmes
The long term risks of invasive breast cancer (IBC) and of death from breast cancer after surgery for DCIS detected by screening are uncertain
More information is needed on how the incidence of IBC after DCIS varies with the characteristics of the patient, the tumour, and the treatment given

**What this study adds**

Following DCIS detected by screening, the rates of invasive breast cancer and of death from breast cancer were more than double the rates expected in the population for at least 20 years
Women who had breast conserving surgery had a lower rate of invasive breast cancer if they also had radiotherapy, and the lowest rate was in women who had mastectomy
Larger surgical margin widths were associated with a lower rate of invasive breast cancer, as was endocrine treatment for women with oestrogen receptor positive disease

## Web extra

Extra material supplied by authors

**Web appendix:** Supplementary material

## Notes

Contributors: GSM, ZW, and SCD contributed equally to this paper. GSM and SCD contributed to the study conceptualisation and design. JB, JC, SC, and OK contributed to data collection, and ZW, GSM, and SCD contributed to data analysis. GSM, SCD, ZW, and DD participated in data interpretation and manuscript preparation. GSM and DD did the literature search. All authors read and approved the final version. The corresponding author attests that all listed authors meet authorship criteria and that no others meeting the criteria have been omitted. GSM is the guarantor.

Ethical approval: This study was approved by the Yorkshire and The Humber-Leeds East Research Ethics Committee (reference: 16/YH/0209), the NHS Breast Screening Programme Research Committee, and Public Health England’s Office for Data Release (reference: ODR1516_225). Informed consent from individual participants was not required.

Data sharing: De-personalised study data may be made available on request to accredited researchers who submit a proposal that is approved by the NHS Breast Screening Programme Research Committee and by the Public Health England Office for Data Release.

The lead author affirms that this manuscript is an honest, accurate, and transparent account of the study being reported; that no important aspects of the study have been omitted; and that any discrepancies from the study as planned (and, if relevant, registered) have been explained.

Dissemination to participants and related patient and public communities: The results have already been presented to representatives of appropriate patient groups. After publication, they will be freely available both to stakeholders and to the broader public.

## References

[1] Bleyer A Welch HG Effect of three decades of screening mammography on breast-cancer incidence. N Engl J Med 2012;367:1998-2005. 10.1056/NEJMoa1206809 23171096

[2] Kerlikowske K Epidemiology of ductal carcinoma in situ. J Natl Cancer Inst Monogr 2010;2010:139-41. . 10.1093/jncimonographs/lgq027 20956818 PMC5161058

[3] Benson JR Wishart GC Predictors of recurrence for ductal carcinoma in situ after breast-conserving surgery. Lancet Oncol 2013;14:e348-57. 10.1016/S1470-2045(13)70135-9 23896274

[4] Virnig BA Tuttle TM Shamliyan T Kane RL Ductal carcinoma in situ of the breast: a systematic review of incidence, treatment, and outcomes. J Natl Cancer Inst 2010;102:170-8. 10.1093/jnci/djp482 20071685

[5] Youngwirth LM Boughey JC Hwang ES Surgery versus monitoring and endocrine therapy for low-risk DCIS: The COMET Trial. Bull Am Coll Surg 2017;102:62-3. 28925613

[6] Francis A Fallowfield L Rea D The LORIS Trial: Addressing overtreatment of ductal carcinoma in situ. Clin Oncol (R Coll Radiol) 2015;27:6-8. 10.1016/j.clon.2014.09.015 25445552

[7] Elshof LE Tryfonidis K Slaets L Feasibility of a prospective, randomised, open-label, international multicentre, phase III, non-inferiority trial to assess the safety of active surveillance for low risk ductal carcinoma in situ - The LORD study. Eur J Cancer 2015;51:1497-510. 10.1016/j.ejca.2015.05.008 26025767

[8] Elshof LE Schaapveld M Schmidt MK Rutgers EJ van Leeuwen FE Wesseling J Subsequent risk of ipsilateral and contralateral invasive breast cancer after treatment for ductal carcinoma in situ: incidence and the effect of radiotherapy in a population-based cohort of 10,090 women [correction: *Breast Cancer Res Treat* 2017;161:389-90]. Breast Cancer Res Treat 2016;159:553-63. 10.1007/s10549-016-3973-y 27624164 PMC5021731

[9] Román M Rué M Sala M Cumulative False Positive Risk Group Trends in detection of invasive cancer and ductal carcinoma in situ at biennial screening mammography in Spain: a retrospective cohort study. PLoS One 2013;8:e83121. 10.1371/journal.pone.0083121 24376649 PMC3871523

[10] Duffy SW Dibden A Michalopoulos D Screen detection of ductal carcinoma in situ and subsequent incidence of invasive interval breast cancers: a retrospective population-based study. Lancet Oncol 2016;17:109-14. 10.1016/S1470-2045(15)00446-5 26655422 PMC4691349

[11] Rakovitch E Nofech-Mozes S Narod SA Can we select individuals with low risk ductal carcinoma in situ (DCIS)? A population-based outcomes analysis. Breast Cancer Res Treat 2013;138:581-90. 10.1007/s10549-013-2455-8 23456231

[12] Thompson AM Clements K Cheung S Sloane Project Steering Group (NHS Prospective Study of Screen-Detected Non-invasive Neoplasias) Management and 5-year outcomes in 9938 women with screen-detected ductal carcinoma in situ: the UK Sloane Project. Eur J Cancer 2018;101:210-9. 10.1016/j.ejca.2018.06.027 30092498

[13] Wapnir IL Dignam JJ Fisher B Long-term outcomes of invasive ipsilateral breast tumor recurrences after lumpectomy in NSABP B-17 and B-24 randomized clinical trials for DCIS. J Natl Cancer Inst 2011;103:478-88. 10.1093/jnci/djr027 21398619 PMC3107729

[14] Cuzick J Sestak I Pinder SE Effect of tamoxifen and radiotherapy in women with locally excised ductal carcinoma in situ: long-term results from the UK/ANZ DCIS trial. Lancet Oncol 2011;12:21-9. 10.1016/S1470-2045(10)70266-7 21145284 PMC3018565

[15] Correa C McGale P Taylor C Early Breast Cancer Trialists’ Collaborative Group (EBCTCG) Overview of the randomized trials of radiotherapy in ductal carcinoma in situ of the breast. J Natl Cancer Inst Monogr 2010;2010:162-77. 10.1093/jncimonographs/lgq039 20956824 PMC5161078

[16] Public Health England. Breast screening: programme overview 2015. https://www.gov.uk/topic/population-screening-programmes/breast.

[17] National Cancer Intelligence Network. About the National Cancer Registration and Analysis Service London: Public Health England; 2010. http://www.ncin.org.uk/about_ncin/.

[18] Breslow NE Day NE Gart JJ Statistical methods in cancer research. World Heath Organization, 1980.

[19] Putter H Fiocco M Geskus RB Tutorial in biostatistics: competing risks and multi-state models. Stat Med 2007;26:2389-430. 10.1002/sim.2712 17031868

[20] Ponti A Ronco G Lynge E ICSN DCIS Working Group Low-grade screen-detected ductal carcinoma in situ progresses more slowly than high-grade lesions: evidence from an international multi-centre study. Breast Cancer Res Treat 2019;177:761-5. 10.1007/s10549-019-05333-6 31250357

[21] Narod SA Iqbal J Giannakeas V Sopik V Sun P Breast Cancer Mortality After a Diagnosis of Ductal Carcinoma In Situ. JAMA Oncol 2015;1:888-96. 10.1001/jamaoncol.2015.2510 26291673

[22] National Institute of Clinical Excellence. Early and locally advanced breast cancer: diagnosis and management NICE guideline [NG101] 2018. https://www.nice.org.uk/guidance/ng101. 35263062

[23] Marinovich ML Azizi L Macaskill P The association of surgical margins and local recurrence in women with ductal carcinoma in situ treated with breast-conserving therapy: a meta-analysis. Ann Surg Oncol 2016;23:3811-21. 10.1245/s10434-016-5446-2 27527715 PMC5160992

[24] Visser LL Groen EJ van Leeuwen FE Lips EH Schmidt MK Wesseling J Predictors of an Invasive Breast Cancer Recurrence after DCIS: A Systematic Review and Meta-analyses. Cancer Epidemiol Biomarkers Prev 2019;28:835-45. 10.1158/1055-9965.EPI-18-0976 31023696

[25] Tadros AB Smith BD Shen Y Ductal Carcinoma In Situ and Margins <2 mm: Contemporary Outcomes With Breast Conservation. Ann Surg 2019;269:150-7. 10.1097/SLA.0000000000002439 28742682 PMC6051916

[26] The Trustees of the Association of Breast Surgery. CONSENSUS STATEMENT: margin width in breast conservation surgery Bournemouth, UK: Association of Breast Surgery; 2015. https://associationofbreastsurgery.org.uk/media/64245/final-margins-consensus-statement.pdf.

[27] Cheung S Booth ME Kearins O Dodwell D Risk of subsequent invasive breast cancer after a diagnosis of ductal carcinoma in situ (DCIS). Breast 2014;23:807-11. 10.1016/j.breast.2014.08.013 25270228

[28] Kanbayashi C Thompson AM Hwang E-SS The international collaboration of active surveillance trials for low-risk DCIS (LORIS, LORD, COMET, LORETTA). J Clin Oncol 2019;37(15_suppl):TPS603 10.1200/JCO.2019.37.15_suppl.TPS603

