# Supplementary material for: Invasive breast cancer and breast cancer mortality after ductal carcinoma in situ in women attending for breast screening in England, 1988-2014: population based observational cohort study
Source: BMJ. 2020 May 27;369:m1570. doi: 10.1136/bmj.m1570 (PMC7251423; doi:10.1136/bmj.m1570)
Supplement: Supplementary file 1 — Web appendix: Supplementary material [file mang051452.ww1.pdf]

Invasive breast cancer and breast cancer mortality after ductal carcinoma in situ in women attending for breast screening in England, 1988-2014: population based observational cohort study

Supplementary Material

Contents

Supplementary Text, Tables and Figures are listed in the order they are referred to in the paper

	Page
**Supplementary Text S1. Statistical methods**	5
**Supplementary Table S1. Missing values in the characteristics of 29,044 women in England diagnosed with DCIS as a result of screening between April 2000 and March 2014.**	10
**Supplementary Table S2. Numbers of women according to number of characteristics missing among the 29,044 women in England diagnosed with DCIS as a result of screening between April 2000 and March 2014.**	11
**Supplementary Table S3. Person-years at risk and numbers of observed and expected deaths from all causes by age at DCIS diagnosis and time since DCIS diagnosis in 35,024 women with DCIS detected as a result of screening between January 1988 and March 2014.**	12
**Supplementary Table S4. Numbers of observed and expected deaths from all causes by calendar period of DCIS diagnosis and time since DCIS diagnosis in 35,024 women with DCIS detected as a result of screening between January 1988 and March 2014.**	13
**Supplementary Table S5. Person-years at risk and numbers of observed and expected invasive breast cancers by age at DCIS diagnosis and time since DCIS diagnosis in 35,024 women with DCIS detected as a result of screening between January 1988 and March 2014.**	14
**Supplementary Table S6. Numbers of observed and expected invasive breast cancers by calendar period of DCIS diagnosis and time since DCIS diagnosis in 35,024 women with DCIS detected as a result of screening between January 1988 and March 2014.**	15

Supplementary Table S7. Numbers of person-years at risk, numbers of observed and expected invasive breast cancers, and cumulative risks by date of DCIS diagnosis and time since DCIS diagnosis in 35,024 women with DCIS detected as a result of screening between January 1988 and March 2014. 16

Supplementary Table S8. Person-years at risk and numbers of observed and expected invasive breast cancers by surgery, whether both breasts were affected and time since diagnosis in 30,559 women with DCIS detected as a result of screening between April 2000 and March 2014. 17

Supplementary Table S9. Person-years at risk and numbers of observed and expected breast cancer deaths by age at DCIS diagnosis and time since DCIS diagnosis in 35,024 women with DCIS detected as a result of screening between January 1988 and March 2014. 18

Supplementary Table S10. Numbers of observed and expected breast cancer deaths by calendar period of DCIS diagnosis and time since DCIS diagnosis in 35,024 women with DCIS detected as a result of screening between January 1988 and March 2014. 18

Supplementary Table S11. Numbers of person-years at risk, numbers of observed and expected breast cancer deaths, and cumulative risks by date of DCIS diagnosis and time since DCIS diagnosis in 35,024 women with DCIS detected as a result of screening between January 1988 and March 2014. 19

Supplementary Table S12. Person-years at risk and numbers of observed and expected breast cancer deaths by surgery, whether both breasts were affected and time since diagnosis in 30,559 women with DCIS detected as a result of screening between April 2000 and March 2014. 20

Supplementary Table S13. Characteristics and vital status on 31 December 2014 of 24,779 women diagnosed with unilateral DCIS detected as a result of screening between April 2000 and March 2014 and who received surgery. 21

Supplementary Figure S1. Incidence of ipsilateral invasive breast cancer according to various factors in 24,779 women diagnosed with unilateral DCIS as a result of screening between April 2000 and March 2014 and who received surgery showing final margin distances of 1 and 2 mm separately. 22

Supplementary Figure S2. Incidence of ipsilateral invasive breast cancer according to various factors in 24,779 women diagnosed with unilateral DCIS detected as a result of screening between April 2000 and March 2014 and who received surgery. 23

Supplementary Figure S3. Incidence of ipsilateral invasive breast cancer according to various factors in 24,779 women diagnosed with unilateral DCIS detected as a result of screening between April 2000 and March 2014 and who received surgery showing final margin distances of 1 and 2 mm separately. 24

Supplementary Table S14. Tests for interactions between pairs of factors for analysis shown in Figure 3. 25

Supplementary Figure S4. Cumulative incidence rate of ipsilateral invasive breast cancer in 24,779 women diagnosed with unilateral DCIS detected as a result of screening between April 2000 and March 2014 by treatment and DCIS grade. 26

Supplementary Figure S5. Annual incidence rate of ipsilateral invasive breast cancer in 24,779 women diagnosed with unilateral DCIS detected as a result of screening between April 2000 and March 2014 and who received surgery by treatment and age at diagnosis of DCIS. 27

Supplementary Figure S6. Incidence rate of contralateral invasive breast cancer according to various factors in 24,779 women diagnosed with unilateral DCIS detected as a result of screening between April 2000 and March 2014 and who received surgery. 28

Supplementary Figure S7. Comparison of the rates of ipsilateral and contralateral invasive breast cancer according to various factors in 24,779 women with unilateral screen-detected DCIS detected as a result of screening between April 2000 and March 2014 and who received surgery. 29

Supplementary Table S15. Characteristics of 29,044 women diagnosed with unilateral DCIS detected as a result of screening between April 2000 and March 2014 and who received surgery, according to oestrogen-receptor status and whether or not they were recorded as having received endocrine therapy. 30

Supplementary Figure S8. Incidence of ipsilateral and contralateral invasive breast cancer in 29,044 women with unilateral screen-detected DCIS according to oestrogen-receptor status and endocrine therapy. 31

Supplementary Figure S9. Incidence of ipsilateral and contralateral invasive breast cancer in 29,044 women with unilateral screen-detected DCIS who underwent surgery according to oestrogen-receptor status, endocrine therapy, and whether they received mastectomy, breast conserving surgery with radiotherapy, or breast conserving surgery without radiotherapy. 32

Supplementary Text S1. Statistical Methods

A Tabulation of person-years at risk and observed events

The analyses were conducted using the standard epidemiological approach of considering the length of time during which a woman was at risk of experiencing an event of interest (invasive breast cancer, invasive ipsilateral breast cancer, invasive contralateral breast cancer, breast cancer death, or death from any cause). The methods described here are for invasive breast cancer (IBC). The methods for the other endpoints are similar.

For each woman who was eligible for the study, the length of time from 6 months after her DCIS diagnosis until the earliest of IBC diagnosis, death, emigration, or 31st December 2014 was calculated. These lengths of time were added together and formed the person-years at risk. These were assumed fixed in the analyses. The number of women whose contribution to the person-years was terminated by an IBC occurring was also obtained. For each analysis both the person-years and the number of IBCs were then tabulated according to the factors needed for that particular analysis.

B Observed and expected rates and ratios of observed to expected rates (Supplementary Tables S3- S12, Supplementary Figure S5)

Observed rates per 1000 person-years were calculated by tabulating the IBCs and the person-years according to categories of the factor of interest (e.g. Age at DCIS diagnosis or Time since DCIS diagnosis) and then for each category dividing the number of IBCs by the number of person-years and multiplying by 1000. The categories for age at DCIS diagnosis were chosen by subdividing the women into 4 groups of approximately equal size whilst remaining close to the age-boundaries used in the screening program while the categories of time since diagnosis were chosen after tabulating the rates in finer time categories to ensure that no features of the data would be concealed by the categorisation. Confidence intervals for observed rates were calculated by assuming that the numbers of IBCs in each category had a Poisson distribution.

Calculation of the numbers of IBCs expected in the study population were based on IBC incidence rates for women in the entire population of England and Wales in five-year groups of attained age for each individual calendar year from 1988-2014. These were provided by Public Health England. The person-years for the study population were then tabulated by these same categories (i.e. five-year groups of attained age within each individual calendar year) as well as for the factors of interest for each specific analysis (e.g. Age at DCIS diagnosis, Time since DCIS diagnosis, Calendar year of DCIS diagnosis). Within each combination of all the tabulated factors, the number of person-years was then multiplied by the relevant attained-age and calendar year specific IBC incidence rate for England and Wales. This provided the number of IBCs expected for that combination of factors. The expected IBCs were then summed over all the factors that were not of direct interest in that analysis (e.g. in Supplementary Table S5 attained age, calendar year of DCIS diagnosis and either Age at DCIS diagnosis or Time since DCIS diagnosis) to provide the number of IBCs expected subdivided by categories of the factor(s) that were of interest in the analysis. For each of these categories, the ratio of observed to expected IBCs (sometimes referred to as the Standardised Incidence Ratio) was then calculated by dividing the number of IBCs observed by the number expected. The confidence intervals for the ratios of observed to expected IBCs were calculated assuming that the number of IBCs observed had a Poisson distribution while the number expected was fixed.

continued overleaf

C **Cumulative observed risk of IBC, accounting for competing risks from causes of death other than breast cancer (Figure 2, Supplementary Tables S7 & S11)**

To derive the cumulative risk that a woman in the study population would develop IBC at 1, 2, 3, …, 18, 19, 20 years after her diagnosis of DCIS, it is necessary to take into account the fact that she may, during this period, die from a cause other than breast cancer before any IBC has had time to develop, thereby reducing the risk that she will develop IBC compared with that suggested just by consideration of the age-specific IBC. The calculation was carried out as follows.

First we divided the time since diagnosis of DCIS into time intervals. The first time interval was of length six months (to account for the fact that women entered the study six months after their diagnosis of DCIS) and subsequent time intervals had length one year. We denote the annual IBC rate (i.e. the observed number of IBCs divided by the person-years) during the tth interval by $r_{ibc,t}$ and the annual competing cause death (CCD) rate during the tth interval by $r_{ccd,t}$.

Based on the standard theory of Poisson processes, the probability that a woman who has survived up to the beginning of time interval t without developing IBC will develop IBC during interval t is:

$$p_{ibc,t} = 1\text{-}\exp[- r_{ibc,t}\, T_t] \,, \qquad (1)$$

where T_t is the length of time interval t in years, while the probability that a woman who has survived up to the beginning of time interval t will die from a CCD during interval t is:

$$p_{ccd,t} = 1\text{-}\exp[- r_{ccd,t}\, T_t] \,,$$

and the probability that a woman who has survived up to the beginning of time interval t without developing IBC will either develop IBC or die from a CCD during interval t is:

$$p_{ibc+ccd,t} = 1\text{-}\exp[- (r_{ibc,t} + r_{ccd,t})T_t],$$

and the probability that she will experience neither event during interval t is:

$$1\text{-}p_{ibc+ccd,t} \,.$$

The probability that a woman will survive up to the end of interval (t-1) without developing IBC can then be calculated recursively by the formula:

$$Q_{ibc+ccd,t-1} = Q_{ibc+ccd,t-2}\,(1\text{-}p_{ibc+ccd,t-1}) \,, \qquad (2)$$

where $Q_{ibc+ccd,0}$ is set to 1.

It then follows that, by combining (1) and (2) above, the probability that a woman will develop an IBC during interval t, accounting for the competing risk of dying previously from another cause is:

$$Q_{ibc+ccd,t-1}\,[1\text{-}\exp(- r_{ibc,t}\, T_t)]. \qquad (3)$$

For each interval, t, the quantities in (3) can then be summed over previous time intervals to obtain the cumulative probability of developing an IBC by the end of interval t. This value can then be multiplied by 100 to provide the corresponding risk in terms of percent. The confidence intervals for the cumulative observed risk of IBC were calculated assuming that the number of IBCs observed had a Poisson distribution.

continued overleaf

D **Cumulative expected risk of IBC, accounting for competing risks from causes of death other than breast cancer (Figure 2, Supplementary Tables S7 & S11)**

The calculation of the cumulative expected risk accounting for competing risks from causes of death other than breast cancer was calculated in a similar fashion to that for the observed risk derived above, except that rather than using the observed annual IBC rate (i.e. the observed number of IBCs divided by the person-years) we used the expected rates (i.e. the expected number of IBCs divided by the person-years) where the expected number of IBCs had been calculated as described above in the section 'Calculation of numbers of IBCs expected'.

E **Comparability between results for women diagnosed with DCIS in different calendar years (Figure 2, Supplementary Tables S7 & S11)**

Death rates in women in the general population have changed during the period in which the National Health Service Breast Screening Programme (NHSBSP) has been in operation and we wished to find a way of examining whether the cumulative risk of developing breast cancer following a diagnosis of screen-detected DCIS had changed that took account of competing causes of death but was not heavily influenced by changes over calendar period in the age-specific death rates from causes other than breast cancer during the study period. Therefore, rather than using individual calendar-year specific values for the CCD rates, we instead used the 2014 death rates for all causes other than breast cancer for England and Wales to account for competing causes of death throughout. This enabled us to be sure that any changes in the cumulative risks of IBC for women diagnosed with DCIS in different calendar years would be attributable to changes in the rate of development of IBC rather than changes in the death rate over calendar time, whilst still taking account of deaths from competing causes.

F **Multiple imputation for missing data on some characteristics (Figures 3-6, Supplementary Figures S1-S9, Supplementary Tables S13-S15)**

Information was available for all the women in the study for dates of screening and DCIS diagnosis, age at DCIS diagnosis, region of residence and treatment, together with dates of emigration, IBC, or death, if any of these occurred before the end of follow-up. For 9,537 of the 29,044 (32.8%) of the women diagnosed between April 2000 and March 2014, information was also available on all the remaining characteristics (DCIS size, DCIS grade, oestrogen-receptor (ER) status, final margin distance and laterality). However, for 50.1%, 14.1%, 2.6%, and 0.4% of women respectively information was missing on 1, 2, 3 or 4 of these characteristics (Tables S1 and S2).

For analyses requiring adjustments, in order to be able to include all the women in every analysis in an appropriate way, irrespective of whether data on some characteristics were missing, the method of multiple imputation was used (Rubin DB, 1987; van Buuren S, 2018). In this method, multiple datasets are created in which the missing values are replaced by imputed (i.e. predicted) values that have been sampled from their predictive distributions. Hence, as well as providing estimates, the method is able to take into account the uncertainty arising from the imputed values as well as the uncertainty in the estimates in any ensuing confidence intervals and significance tests.

All the variables for which any values were missing were categorical and there were no complex design features in the data. For each missing value, its predictive distribution was obtained using chained multinomial logistic regressions for the variables for which some values were missing, starting with the variable for which there were fewest missing values. Additional independent variables in the predictions were year of DCIS diagnosis, age at DCIS diagnosis, region, treatment, invasive breast cancer, and death from breast cancer.

continued overleaf

Calculations involving multiple imputation were carried out using the multiple imputation suite of programs in Stata [StataCorp]. The default burn-in period was 10 iterations and, to confirm that this number was sufficient, trace plots for a burn-in period of 100 iterations were produced and examined. To examine the plausibility of the imputed values, the distributions of the imputed and recorded values for each variable were compared and in every case good agreement was found. Results presented in the paper are based on a total of 60 imputed datasets. This number was chosen because the largest percentage missing for any variable was 53.3%, for ER-status.

For the variables where imputation was necessary, the numbers of women given in each category in the tables in this report are the numbers of women averaged over the imputed datasets. For analyses requiring adjustments, each analysis was carried out separately on every imputed dataset, as described in sections **G. H**. and **I**. below. The resulting estimates and their variances were then combined via Rubin's rules [Rubin, 1987].

Every analysis involving multiple imputation was also carried out on the original dataset with any missing values assigned to a separate category, rather than imputed. In every case, the results from the two different approaches were virtually identical.

G Variation in incidence of ipsilateral IBC rate (or the BCD rate) with patient and tumour characteristics and treatment (Figure 3, Supplementary Figures S1-S3 & S6-S9)

The extent to which the incidence of ipsilateral IBC (iIBC) varied with the patient and tumour characteristics and the treatment given (e.g. Figure 3) was studied using Poisson regression. To do this both the numbers of iIBCs and the relevant person-years were tabulated according to all the factors shown in Figure 3, and for each factor the categories shown in that figure were used. The boundaries of these categories were chosen by tabulating each variable individually to have as many categories as needed in order to preserve the features of the data whilst avoiding categories with very little data. The numbers of iIBCs in each cell of the table were assumed to have a Poisson distribution and, to study the variation in one particular characteristic, the number of iIBCs expected in the jth cell of the table were assumed to be given by:

$$\exp(y_j+\mu+\beta_1x_1+\beta_2x_2+\ldots.\beta_kx_k) \qquad (4)$$

where y_j is the number of person-years in the cell, μ is an unknown constant common to all cells, $x_1, x_2, \ldots, x_k$ are indicator variables denoting the different categories of the factor under study, β_1 is assumed to be 0 (i.e. setting the first category of the factor as the baseline category), and $\beta_2, \ldots, \beta_k$ are unknown parameters. Models of this form were fitted to the data by the method of maximum likelihood and the results are shown in the left-hand panel of Figure 3, where the values displayed are the estimates of the $\exp(\beta_i)$ (which correspond to rate ratios) with confidence intervals estimated by the standard errors of the $\exp(\beta_i)$. The models in the right-hand side of Figure 3 were similar, but in this case additional terms representing all the other factors shown in Figure 3 (apart from final margin distance, where information was available only from 2007 onwards) were also included in the model.

continued overleaf

H Significance tests for heterogeneity or trend in the rate ratios for different levels of a factor and in interactions between two factors (Figure 3, Supplementary Figures S1-S3, Supplementary Table 12 , Supplementary Figures S6-S9)

Significance tests for heterogeneity between the rate ratios for the different levels of a factor were based on models of the form (4) in the previous section. For analyses involving multiple imputation they were carried out using the Wald test and assuming that the between-imputation variance was proportional to the within-imputation variance, while for other analyses they used the likelihood ratio. Where the rate ratio involved the number of IBCs expected from national cancer incidence rates, rather than the number of IBCs in the baseline category, y_j was replaced by the number of IBCs expected in cell j. Where there was a natural ordering to the factor of interest, then for the significance test the indicator variables in (4) above were replaced by a variable that took values 1, 2, 3, …, k over the different levels of the factor. Significance tests for interactions between the different factors (see Supplementary Table S14) were carried out in a similar fashion with inclusion of terms for the interaction between two variables in models of the form (4) above.

I Cumulative rates (Figures 4, 5, Supplementary Figure 4)

To obtain cumulative rates, the person-years and IBCs were tabulated simultaneously according to categories of time since DCIS diagnosis and of the factor of interest (e.g. for Figure 4a: Breast-conserving surgery with radiotherapy, Breast conserving surgery without radiotherapy, and Mastectomy). For time since exposure the cut-points used to define the categories were chosen after inspection of the observed rates in Supplementary Table S3. For level k of the factor of interest, the cumulative rate in per-cent up to time T is then

$$100\Sigma(O_{ibc,t}/d_t y_t)$$

where $O_{ibc,t}$ is the number of observed events in time category t, y_t is the number of person-years in category t, d_t is the length of category t in years, and summation is over all categories of t up to T. Confidence intervals for cumulative rates were calculated by first assuming that the numbers of IBCs in each category had a Poisson distribution for each individual dataset. In all the figures where cumulative rates are presented (Figure 4, 5, Supplementary Figure S4) checks were made that adjustment for the potential confounding factors made no material difference to the results.

References for Supplementary Text S1:

StataCorp. Stata Statistical Software: Release 15.1 [program] StataCorp, Texas. 2017.

Rubin D. B. Multiple Imputation for Nonresponse in Surveys. John Wiley and Sons, New York. 1987.

Royston, P. and Lambert P.C. Flexible Parametric Survival Analysis Using Stata: Beyond the Cox Model. Stata Press, College Station, TX. 2011.

Supplementary Table S1. Missing values in the characteristics of 29,044 women in England diagnosed with DCIS as a result of screening between April 2000 and March 2014.

	BCS + RT	BCS - RT	Mastectomy	Total
**DCIS size (mm)**				
<=10	1523 (28.4)	6487 (42.7)	779 (9.2)	8789 (30.3)
11-20	2057 (38.3)	4450 (29.3)	1547 (18.2)	8054 (27.7)
21-50	1487 (27.7)	2829 (18.6)	3745 (44.1)	8061 (27.8)
51+	72 (1.3)	208 (1.4)	1791 (21.1)	2071 (7.1)
Missing	229 (4.3)	1214 (8.0)	626 (7.4)	2069 (7.1)
**DCIS grade**				
Low/intermediate	1319 (24.6)	7631 (50.2)	2214 (26.1)	11,164 (38.4)
High	3831 (71.4)	6810 (44.8)	5823 (68.6)	16,464 (56.7)
Missing	218 (4.1)	747 (4.9)	451 (5.3)	1416 (4.9)
**Oestrogen receptor status and endocrine therapy**				
ER+, no endocrine	1367 (25.5)	3640 (24.0)	2032 (23.9)	7039 (24.2)
ER+, endocrine	478 (8.9)	2340 (15.4)	775 (9.1)	3593 (12.4)
ER-	524 (9.8)	1230 (8.1)	1184 (13.9)	2938 (10.1)
Missing	2999 (55.9)	7978 (52.5)	4497 (53.0)	15,474 (53.3)
**Laterality of DCIS**				
Left	2797 (52.1)	7767 (51.1)	4349 (51.2)	14,913 (51.3)
Right	2562 (47.7)	7314 (48.2)	4108 (48.4)	13,984 (48.1)
Missing	9 (0.2)	107 (0.7)	31 (0.4)	147 (0.5)
**Final margin distance (mm)**				
5+	1763 (32.8)	3148 (20.7)	2297 (27.1)	7208 (24.8)
3-4	542 (10.1)	1132 (7.5)	317 (3.7)	1991 (6.9)
1-2	683 (12.7)	1605 (10.6)	535 (6.3)	2823 (9.7)
Involved	77 (1.4)	203 (1.3)	128 (1.5)	408 (1.4)
Before 2007	1184 (22.1)	5885 (38.7)	3223 (38.0)	10,292 (35.4)
Missing	1119 (20.8)	3215 (21.2)	1988 (23.4)	6322 (21.8)
**Total**	5368 (100.0)	15,188 (100.0)	8488 (100.0)	29,044 (100.0)

BCS+RT: breast-conserving surgery, radiotherapy recorded
BCS-RT: breast-conserving surgery, radiotherapy not recorded
ER+, no endocrine: oestrogen-receptor positive DCIS, endocrine therapy not recorded
ER+, endocrine: oestrogen-receptor positive DCIS, endocrine therapy recorded
ER-: oestrogen-receptor negative DCIS

Supplementary Table S2. Numbers of women according to number of characteristics missing among the 29,044 women in England diagnosed with DCIS as a result of screening between April 2000 and March 2014.

Number of characteristics missing	Laterality	DCIS grade	DCIS size	Final margin distance	ER status	Number of women
**None**	-	-	-	-	-	9,537 (32.8%)
**1 missing**						14,549 (50.1%)
	-	-	-	-	m	11,274
	-	-	-	m	-	2,782
	-	m	-	-	-	233
	-	-	m	-	-	219
	m	-	-	-	-	41
**2 missing**						4099 (14.1%)
	-	-	-	m	m	2,676
	-	-	m	-	m	478
	-	m	m	-	-	337
	-	m	-	-	m	236
	-	-	m	m	-	209
	-	m	-	m	-	77
	m	-	-	-	m	74
	m	-	-	m	-	6
	m	-	m	-	-	4
	m	m	-	-	-	2
**3 missing**						755 (2.6%)
	-	-	m	m	m	317
	-	m	m	-	m	268
	-	m	m	m	-	121
	-	m	-	m	m	34
	m	-	m	-	m	11
	m	m	-	-	m	3
	m	m	m	-	-	1
**4 missing**						104 (0.4%)
	-	m	m	m	m	99
	m	m	m	-	m	4
	m	m	m	m	-	1
**Total number of women**						29,044

Supplementary Table S3. Person-years at risk and numbers of observed and expected deaths from all causes by age at DCIS diagnosis and time since DCIS diagnosis in 35,024 women with DCIS detected as a result of screening between January 1988 and March 2014. Expected values are based on mortality rates for England and Wales in single calendar years and five-year age-groups.

	Person-years at risk	Total number of deaths observed	Observed rate per 1000 person-years (95% confidence interval)	Total number of deaths expected	Ratio of observed to expected* (95% confidence interval)
**Age at DCIS diagnosis**					
<55	81,859	396	4.84 (4.38 to 5.34)	422.23	0.94 (0.85 to 1.03)
55-59	60,505	482	7.97 (7.29 to 8.71)	533.71	0.90 (0.83 to 0.99)
60-64	59,421	697	11.73 (10.89 to 12.63)	856.38	0.81 (0.76 to 0.88)
65+	43,302	659	15.22 (14.10 to 16.43)	1070.43	0.62 (0.57 to 0.66)
P for heterogeneity:			*<0.001*		*<0.001*
P for trend:			*<0.001*		*<0.001*
**Time since DCIS diagnosis (years)**					
5-	121,250	655	5.40 (5.00 to 5.83)	1026.38	0.64 (0.59 to 0.69)
10-	79,072	762	9.64 (8.98 to 10.35)	949.09	0.80 (0.75 to 0.86)
15+	32,512	502	15.44 (14.15 to 16.85)	544.85	0.92 (0.84 to 1.01)
15+	12,253	315	25.71 (23.02 to 28.71)	362.43	0.87 (0.78 to 0.97)
P for heterogeneity:			*<0.001*		*<0.001*
P for trend:			*<0.001*		*<0.001*
**Total**	245,087	2234	9.12 (8.74 to 9.50)	2882.75	0.77 (0.74 to 0.81)
P for test of observed/expected = 1:					*<0.001*

*i. e. estimated ratio of observed rate to expected rate. See supplementary text 1 for further details

Supplementary Table S4. Numbers of observed and expected deaths from all causes by calendar period of DCIS diagnosis and time since DCIS diagnosis in 35,024 women with DCIS detected as a result of screening between January 1988 and March 2014. Expected values are based on mortality rates for England and Wales in single calendar years and five-year age-groups.

Time since DCIS diagnosis (years)	Year of diagnosis of DCIS											
	1988-1999			2000-2004			2005-2009			2010-2014		
	**Obs**	**Exp**	**Ratio* (95%CI)**	**Obs**	**Exp**	**Ratio (95%CI)**	**Obs**	**Exp**	**Ratio (95%CI)**	**Obs**	**Exp**	**Ratio (95%CI)**
0.5-	105	156.15	0.67 (0.56 to 0.81)	177	256.97	0.69 (0.59 to 0.80)	263	426.94	0.62 (0.55 to 0.70)	110	186.32	0.59 (0.49 to 0.71)
5-	211	237.29	0.89 (0.78 to 1.02)	310	391.9	0.79 (0.71 to 0.88)	241	319.91	0.75 (0.66 to 0.85)			
10-	294	324.33	0.91 (0.81 to 1.02)	208	220.52	0.94 (0.82 to 1.08)						
15+	315	362.43	0.87 (0.78 to 0.97)									
**Total**	925	1080.2	0.86 (0.80 to 0.91)	695	869.38	0.80 (0.74 to 0.86)	504	746.85	0.67 (0.62 to 0.74)	110	186.32	0.59 (0.49 to 0.71)

P for trend with year of diagnosis: <0.001 (unadjusted), 0.50 (adjusted for time since diagnosis and age at diagnosis)

Obs: total number of deaths observed
Exp: total number of deaths expected
CI: confidence interval
*i. e. estimated ratio of observed rate to expected rate. See supplementary text 1 for further details

Supplementary Table S5. Person-years at risk and numbers of observed and expected invasive breast cancers by age at DCIS diagnosis and time since DCIS diagnosis in 35,024 women with DCIS detected as a result of screening between January 1988 and March 2014. Expected values are based on cancer incidence rates for England in single calendar years and five-year age-groups.

	Person-years at risk	Number of invasive breast cancers observed	Observed rate per 1000 person-years (95% confidence interval)	Number of invasive breast cancers expected	Ratio of observed to expected* (95% confidence interval)
**Age at DCIS diagnosis**					
<55	78,542	683	8.70 (8.07 to 9.37)	247.17	2.76 (2.56 to 2.98)
55-59	57,775	507	8.78 (8.04 to 9.57)	204.32	2.48 (2.27 to 2.71)
60-64	56,958	504	8.85 (8.11 to 9.66)	212.75	2.37 (2.17 to 2.59)
65+	42,125	382	9.07 (8.20 to 10.0)	160.35	2.38 (2.16 to 2.63)
P for heterogeneity:			*0.93*		*0.03*
P for trend:			*0.52*		*0.007*
**Time since DCIS diagnosis (years)**					
0.5-	16,264	56	3.44 (2.65 to 4.47)	53.00	1.06 (0.81 to 1.37)
1-	30,086	211	7.01 (6.13 to 8.03)	98.89	2.13 (1.86 to 2.44)
2-	27,041	240	8.88 (7.82 to 10.1)	90.05	2.67 (2.35 to 3.02)
3-	46,172	448	9.70 (8.84 to 10.6)	157.06	2.85 (2.60 to 3.13)
5-	36,842	347	9.42 (8.48 to 10.5)	129.30	2.68 (2.42 to 2.98)
7-	38,368	394	10.3 (9.30 to 11.3)	139.57	2.82 (2.56 to 3.12)
10-	29,781	283	9.50 (8.46 to 10.7)	113.40	2.50 (2.22 to 2.80)
15-	9015	77	8.54 (6.83 to 10.7)	35.61	2.16 (1.73 to 2.70)
20+	1830	20	10.9 (7.05 to 16.9)	7.71	2.59 (1.67 to 4.02)
P for heterogeneity:			*<0.001*		*<0.001*
P for trend excluding 1st 3 years:			*<0.001*		*0.53*
**Total**	235,400	2076	8.82 (8.45 to 9.21)	824.59	2.52 (2.41 to 2.63)
P for test of observed/expected = 1:					*<0.001*
**Total 3+ years**	162,009	1569	9.68 (9.22 to 10.2)	582.65	2.69 (2.56 to 2.83)
P for test of observed/expected 3+ years= 1:					*<0.001*

*i. e. estimated ratio of observed rate to expected rate. See supplementary text 1 for further details

Supplementary Table S6. Numbers of observed and expected invasive breast cancers by calendar period of DCIS diagnosis and time since DCIS diagnosis in 35,024 women with DCIS detected as a result of screening between January 1988 and March 2014. Expected values are based on cancer incidence rates for England in single calendar years and five-year age-groups.

**Time since DCIS diagnosis (years)**	**Year of diagnosis of DCIS**											
	**1988-1999**			**2000-2004**			**2005-2009**			**2010-2014**		
	**Obs**	**Exp**	**Ratio* (95%CI)**	**Obs**	**Exp**	**Ratio (95%CI)**	**Obs**	**Exp**	**Ratio (95%CI)**	**Obs**	**Exp**	**Ratio (95%CI)**
0.5-	12	5.55	2.16 (1.23 to 3.80)	14	11.67	1.20 (0.71 to 2.03)	15	18.06	0.83 (0.50 to 1.38)	15	17.71	0.85 (0.51 to 1.40)
1-	35	11.34	3.09 (2.22 to 4.30)	46	23.46	1.96 (1.47 to 2.62)	73	35.90	2.03 (1.62 to 2.56)	57	28.18	2.02 (1.56 to 2.62)
2-	32	11.67	2.74 (1.94 to 3.88)	62	23.50	2.64 (2.06 to 3.38)	90	35.64	2.52 (2.05 to 3.10)	56	19.24	2.91 (2.24 to 3.78)
3-	76	24.65	3.08 (2.46 to 3.86)	128	46.89	2.73 (2.30 to 3.25)	191	70.88	2.69 (2.34 to 3.11)	53	14.65	3.62 (2.76 to 4.74)
5-	71	25.80	2.75 (2.18 to 3.47)	109	46.11	2.36 (1.96 to 2.85)	167	57.38	2.91 (2.50 to 3.39)			
7-	121	38.96	3.11 (2.60 to 3.71)	192	68.71	2.79 (2.43 to 3.22)	81	31.9	2.54 (2.04 to 3.16)			
10-	167	63.33	2.64 (2.27 to 3.07)	116	50.06	2.32 (1.93 to 2.78)						
15-	77	35.61	2.16 (1.73 to 2.70)									
20+	20	7.71	2.59 (1.67 to 4.02)									
**Total**	611	224.62	2.72 (2.51 to 2.94)	667	270.41	2.47 (2.29 to 2.66)	617	249.77	2.47 (2.28 to 2.67)	181	79.78	2.27 (1.96 to 2.62)

P for trend with year of diagnosis: 0.03 (unadjusted), 0.26 (adjusted for time since diagnosis and age at diagnosis)

Obs: number of cancers observed
Exp: number of cancers expected
CI: confidence interval
*i. e. estimated ratio of observed rate to expected rate. See supplementary text 1 for further details

Supplementary Table S7. Numbers of person-years at risk, numbers of observed and expected invasive breast cancers, and cumulative risks by date of DCIS diagnosis and time since DCIS diagnosis in 35,024 women with DCIS detected as a result of screening between January 1988 and March 2014. Expected values are based on cancer incidence rates for England in single calendar years and five-year age-groups

Date of diagnosis of DCIS	Time since DCIS diagnosis (years)	Number of person-years	Number of invasive breast cancers observed	Cumulative observed risk*	95% confidence interval for cumulative observed risk*	Number of invasive breast cancers expected*	Cumulative expected risk*
1988-1999	0.5-	2081	12	0.3	0.1 to 0.5	5.6	0.1
	1-	4125	35	1.1	0.8 to 1.4	11.3	0.4
	2-	4076	32	1.9	1.5 to 2.3	11.7	0.7
	3-	4024	33	2.7	2.2 to 3.2	12.1	1.0
	4-	3962	43	3.7	3.1 to 4.3	12.5	1.3
	5-	3899	35	4.6	3.9 to 5.2	12.8	1.6
	6-	3827	36	5.4	4.7 to 6.1	13.0	1.9
	7-	3758	37	6.3	5.6 to 7.1	13.0	2.3
	8-	3687	45	7.4	6.6 to 8.2	13.0	2.6
	9-	3611	39	8.3	7.5 to 9.2	13.0	2.9
	10-	3534	43	9.4	8.5 to 10.3	12.9	3.2
	11-	3456	28	10.0	9.1 to 11.0	12.7	3.6
	12-	3382	32	10.8	9.9 to 11.8	12.6	3.9
	13-	3317	16	11.2	10.2 to 12.1	12.6	4.2
	14-	3241	48	12.3	11.3 to 13.3	12.6	4.6
	15-	2839	22	12.9	11.9 to 13.9	11.2	4.9
	16-	2221	17	13.5	12.4 to 14.5	8.8	5.2
	17-	1718	10	13.9	12.8 to 15.0	6.8	5.5
	18-	1280	18	14.9	13.7 to 16.0	5.1	5.8
	19+	2786	30	15.6†	14.3 to 16.8	11.5	6.1†
2000-2004	0.5-	3516	14	0.2	0.1 to 0.3	11.7	0.2
	1-	6987	46	0.9	0.6 to 1.1	23.5	0.5
	2-	6905	62	1.7	1.4 to 2.0	23.5	0.8
	3-	6800	67	2.7	2.3 to 3.1	23.5	1.2
	4-	6702	61	3.5	3.1 to 4.0	23.4	1.5
	5-	6600	61	4.4	3.9 to 4.9	23.2	1.8
	6-	6504	48	5.1	4.6 to 5.6	22.9	2.2
	7-	6414	51	5.8	5.3 to 6.3	22.8	2.5
	8-	6299	91	7.1	6.5 to 7.7	22.8	2.8
	9-	6163	50	7.8	7.2 to 8.4	23.1	3.2
	10-	5265	40	8.4	7.8 to 9.1	20.2	3.5
	11-	3723	47	9.5	8.8 to 10.2	14.5	3.9
	12-	2325	17	10.1	9.3 to 10.8	9.2	4.2
	13-	1208	10	10.7	9.9 to 11.6	4.9	4.5
	14+	331	2	11.2†	10.1 to 12.2	1.4	4.9†
2005-2009	0.5-	5358	15	0.1	0.1 to 0.2	18.1	0.2
	1-	10635	73	0.8	0.7 to 1.0	35.9	0.5
	2-	10507	90	1.7	1.4 to 1.9	35.6	0.8
	3-	10358	101	2.6	2.3 to 2.9	35.4	1.2
	4-	10211	90	3.4	3.1 to 3.8	35.5	1.5
	5-	9075	121	4.7	4.3 to 5.1	32.2	1.8
	6-	6937	46	5.3	4.8 to 5.7	25.2	2.2
	7-	4769	42	6.1	5.6 to 6.5	17.8	2.5
	8-	2762	33	7.1	6.5 to 7.7	10.6	2.9
	9+	906	6	7.7†	6.9 to 8.4	3.6	3.2†
2010-2014	0.5-	5310	15	0.1	0.1 to 0.2	17.7	0.2
	1-	8339	57	0.8	0.6 to 1.0	28.2	0.5
	2-	5553	56	1.8	1.5 to 2.1	19.2	0.8
	3-	3127	34	2.8	2.4 to 3.3	11.1	1.2
	4+	989	19	4.6†	3.7 to 5.6	3.6	1.5†

Cumulative risks take into account competing risks from other causes of death. See supplementary text 1 for further details
*Risks given for the end of each period, i.e. risks in line '9-' refer to 10 years after diagnosis, etc
†Risks at 20, 15, 10, and 5 years after diagnosis of DCIS respectively.

Supplementary Table S8. Person-years at risk and numbers of observed and expected invasive breast cancers by surgery, whether both breasts were affected and time since diagnosis in 30,559 women with DCIS detected as a result of screening between April 2000 and March 2014. Expected values are based on cancer incidence rates for England in single calendar years and five-year age-groups.

	**Person-years at risk**	**Number of invasive breast cancers observed**	**Observed rate per 1000 person-years (95% confidence interval)**	**Number of invasive breast cancers expected**	**Ratio of observed to expected* (95% confidence interval)**
**Less than 3 years since diagnosis of DCIS**					
Unilateral with surgery	59,518	383	6.43 (5.82 to 7.11)	201.31	1.90 (1.72 to 2.10)
Unilateral with no/unknown surgery	2875	37	12.87 (9.32 to 17.8)	9.75	3.80 (2.75 to 5.24)
Bilateral	126	2	15.82 (3.96 to 63.3)	0.43	4.63 (1.16 to 18.5)
P for heterogeneity:			*<0.001*		*<0.001*
Total less than 3 years since diagnosis of DCIS	62,520	422	6.75 (6.14 to 7.43)	211.49	2.00 (1.81 to 2.20)
**At least 3 years since diagnosis of DCIS**					
Unilateral with surgery	98,639	933	9.46 (8.87 to 10.1)	354.54	2.63 (2.47 to 2.81)
Unilateral with no/unknown surgery	6121	75	12.25 (9.77 to 15.4)	22.16	3.38 (2.70 to 4.24)
Bilateral	212	3	14.12 (4.55 to 43.8)	0.76	3.96 (1.28 to 12.30)
P for heterogeneity:			*0.09*		*0.11*
Total at least 3 years since diagnosis of DCIS	104,972	1011	9.63 (9.06 to 10.24)	377.46	2.68 (2.52 to 2.85)
**All years since diagnosis of DCIS**					
Unilateral with surgery	158,157	1316	8.32 (7.88 to 8.78)	555.85	2.37 (2.24 to 2.50)
Unilateral with no/unknown surgery	8996	112	12.45 (10.4 to 15.0)	31.91	3.51 (2.92 to 4.22)
Bilateral	339	5	14.75 (6.14 to 35.5)	1.19	4.21 (1.75 to 10.1)
P for heterogeneity:			*<0.001*		*<0.001*
**Total**	167,492	1433	8.56 (8.12 to 9.01)	588.95	2.43 (2.31 to 2.56)

*i. e. estimated ratio of observed rate to expected rate. See supplementary text 1 for further details

Supplementary Table S9. Person-years at risk and numbers of observed and expected breast cancer deaths by age at DCIS diagnosis and time since DCIS diagnosis in 35,024 women with DCIS detected as a result of screening between January 1988 and March 2014. Expected values are based on mortality rates for England and Wales in single calendar years and five-year age-groups.

	Person-years at risk	Number of breast cancer deaths observed	Observed rate per 1000 person-years (95% confidence interval)	Number of breast cancer deaths expected	Ratio of observed to expected* (95% confidence interval)
**Age at DCIS diagnosis**					
<55	81,859	92	1.12 (0.92 to 1.38)	44.37	2.07 (1.69 to 2.54)
55-59	60,505	78	1.29 (1.03 to 1.61)	42.13	1.85 (1.48 to 2.31)
60-64	59,421	82	1.38 (1.11 to 1.71)	50.21	1.63 (1.32 to 2.03)
65+	43,302	58	1.34 (1.04 to 1.73)	45.38	1.28 (0.99 to 1.65)
P for heterogeneity:			*0.54*		*0.03*
P for trend:			*0.20*		*0.003*
**Time since DCIS diagnosis (years)**					
0.5-	121,250	70	0.58 (0.46 to 0.73)	80.58	0.87 (0.69 to 1.10)
5-	79,072	118	1.49 (1.25 to 1.79)	59.69	1.98 (1.65 to 2.37)
10-	32,512	84	2.58 (2.09 to 3.20)	28.09	2.99 (2.41 to 3.70)
15+	12,253	38	3.10 (2.26 to 4.26)	13.74	2.77 (2.01 to 3.80)
P for heterogeneity:			*<0.001*		*<0.001*
P for trend excluding 1st 5 years:			*<0.001*		*0.01*
**Total**	245,087	310	1.26 (1.13 to 1.41)	182.1	1.70 (1.52 to 1.90)
P for test of observed/expected = 1:					*<0.001*
**Total 5+ years**	123,837	240	1.94 (1.71 to 2.20)	101.52	2.36 (2.08 to 2.68)
P for test of observed/expected 5+ years= 1:					*<0.001*

*i. e. estimated ratio of observed rate to expected rate. See supplementary text 1 for further details

Supplementary Table S10 Numbers of observed and expected breast cancer deaths by calendar period of DCIS diagnosis and time since DCIS diagnosis in 35,024 women with DCIS detected as a result of screening between January 1988 and March 2014. Expected values are based on mortality rates for England and Wales in single calendar years and five-year age-groups.

Time since DCIS diagnosis (years)	Year of diagnosis of DCIS											
	1988-1999			2000-2004			2005-2009			2010-2014		
	Obs	Exp	Ratio* (95%CI)	Obs	Exp	Ratio (95%CI)	Obs	Exp	Ratio (95%CI)	Obs	Exp	Ratio
0.5-	9	14.51	0.62 (0.32 to 1.19)	22	21.55	1.02 (0.67 to 1.55)	32	31.13	1.03 (0.73 to 1.45)	7	13.38	0.52 (0.25 to 1.10)
5-	41	16.47	2.49 (1.83 to 3.38)	50	24.96	2.00 (1.52 to 2.64)	27	18.26	1.48 (1.01 to 2.16)			
10-	58	16.91	3.43 (2.65 to 4.44)	26	11.18	2.32 (1.58 to 3.41)						
15+	38	13.74	2.77 (2.01 to 3.80)									
**Total**	146	61.63	2.37 (2.01 to 2.79)	98	57.7	1.70 (1.39 to 2.07)	59	49.4	1.19 (0.93 to 1.54)	7	13.38	0.52 (0.25 to 1.10)

P for trend with year of diagnosis: *<0.0001 (unadjusted), 0.09 (adjusted for time since diagnosis and age at diagnosis)*

Obs: number of breast cancer deaths observed
Exp: number of breast cancer deaths expected
CI: confidence interval
*i. e. estimated ratio of observed rate to expected rate. See supplementary text 1 for further details

Supplementary Table S11. Numbers of person-years at risk, numbers of observed and expected breast cancer deaths, and cumulative risks by date of DCIS diagnosis and time since DCIS diagnosis in 35,024 women with DCIS detected as a result of screening between January 1988 and March 2014. Expected values are based on mortality rates for England and Wales in single calendar years and five-year age-groups.

Date of diagnosis of DCIS	Time since DCIS diagnosis (years)	Number of person-years	Number of breast cancer deaths observed	Cumulative observed risk*	95% confidence interval for cumulative observed risk*	Number of breast cancer deaths expected*	Cumulative expected risk*
1988-1999	0.5-	2084	1	0	0.00 to 0.04	1.6	0.0
	1-	4156	1	0	0.00 to 0.09	3.2	0.1
	2-	4137	2	0.1	0.03 to 0.19	3.2	0.2
	3-	4113	4	0.2	0.1 to 0.3	3.2	0.3
	4-	4084	1	0.2	0.1 to 0.4	3.2	0.3
	5-	4053	7	0.4	0.2 to 0.6	3.3	0.4
	6-	4011	8	0.6	0.3 to 0.8	3.3	0.5
	7-	3964	10	0.8	0.5 to 1.1	3.3	0.6
	8-	3919	7	1.0	0.7 to 1.3	3.3	0.7
	9-	3879	9	1.2	0.9 to 1.5	3.3	0.7
	10-	3824	13	1.5	1.1 to 1.9	3.4	0.8
	11-	3766	12	1.8	1.4 to 2.2	3.4	0.9
	12-	3709	15	2.2	1.7 to 2.6	3.4	1.0
	13-	3654	3	2.2	1.8 to 2.7	3.4	1.1
	14-	3593	15	2.6	2.1 to 3.1	3.4	1.2
	15-	3174	4	2.7	2.2 to 3.2	3.1	1.2
	16-	2492	8	3.0	2.4 to 3.5	2.6	1.3
	17-	1934	6	3.2	2.7 to 3.8	2.1	1.4
	18-	1458	5	3.5	2.9 to 4.1	1.7	1.5
	19+	3196	15	3.8†	3.2 to 4.5	4.3	1.6†
2000-2004	0.5-	3519	1	0	0.00 to 0.04	2.3	0.0
	1-	7022	2	0	0.00 to 0.09	4.7	0.1
	2-	6991	5	0.1	0.03 to 0.19	4.8	0.2
	3-	6951	6	0.2	0.1 to 0.3	4.8	0.2
	4-	6902	8	0.3	0.2 to 0.4	4.9	0.3
	5-	6851	12	0.5	0.3 to 0.6	4.9	0.4
	6-	6791	10	0.6	0.4 to 0.8	5.0	0.4
	7-	6738	7	0.7	0.5 to 0.9	5.0	0.5
	8-	6681	10	0.9	0.6 to 1.1	5.0	0.6
	9-	6605	11	1.0	0.8 to 1.2	5.1	0.7
	10-	5675	13	1.2	1.0 to 1.5	4.4	0.7
	11-	4036	10	1.4	1.2 to 1.7	3.2	0.8
	12-	2546	1	1.5	1.2 to 1.8	2.1	0.9
	13-	1338	2	1.6	1.3 to 2.0	1.1	0.9
	14+	372	0	1.6†	1.3 to 2.0	0.3	1.0†
2005-2009	0.5-	5360	2	0	0.00 to 0.04	3.4	0.0
	1-	10682	10	0.1	0.05 to 0.18	6.8	0.1
	2-	10631	7	0.2	0.1 to 0.3	6.9	0.2
	3-	10576	8	0.3	0.2 to 0.3	6.9	0.2
	4-	10511	5	0.3	0.2 to 0.4	7.0	0.3
	5-	9428	10	0.4	0.3 to 0.5	6.5	0.4
	6-	7248	5	0.5	0.3 to 0.6	5.2	0.4
	7-	5014	7	0.6	0.4 to 0.8	3.7	0.5
	8-	2925	4	0.7	0.5 to 0.9	2.2	0.6
	9+	966	1	0.8†	0.5 to 1.1	0.7	0.6†
2010-2014	0.5-	5313	0	0	0.00 to 0.00	2.9	0.0
	1-	8382	4	0	0.00 to 0.09	4.7	0.1
	2-	5623	3	0.1	0.02 to 0.18	3.3	0.1
	3-	3189	0	0.1	0.02 to 0.18	1.9	0.2
	4+	1023	0	0.1†	0.02 to 0.18	0.6	0.3†

Cumulative risks take into account competing risks from other causes of death. See supplementary text 1 for further details
*Risks given for the end of each period, i.e. risks in line '9-' refer to 10 years after diagnosis, etc
†Risks at 20, 15, 10, and 5 years after diagnosis of DCIS respectively

Supplementary Table S12. Person-years at risk and numbers of observed and expected breast cancer deaths by surgery, whether both breasts were affected and time since diagnosis in 30,559 women with DCIS detected as a result of screening between April 2000 and March 2014. Expected values are based on mortality rates for England and Wales in single calendar years and five-year age-groups.

	Person-years at risk	Number of breast cancer deaths observed	Observed rate pre 1000 person-years (95% confidence interval)	Number of breast cancer deaths expected	Ratio of observed to expected* (95% confidence interval)
**Less than 5 years since diagnosis of DCIS**					
Unilateral with surgery	96,650	52	0.54 (0.41 to 0.71)	62.05	0.84 (0.64 to 1.10)
Unilateral with no/unknown surgery	4748	9	1.90 (0.99 to 3.64)	3.15	2.86 (1.49 to 5.49)
Bilateral	207	0	-	0.13	-
P for heterogeneity:			*0.009*		*0.01*
Total less than 5 years since diagnosis of DCIS	101,605	61	0.60 (0.47 to 0.77)	35.33	0.93 (0.73 to 1.20)
**At least 5 years since diagnosis of DCIS**					
Unilateral with surgery	66,169	84	1.27 (1.03 to 1.57)	48.97	1.72 (1.38 to 2.12)
Unilateral with no/unknown surgery	4666	14	3.00 (1.78 to 5.07)	3.6	3.89 (2.31 to 6.57)
Bilateral	141	1	7.07 (1.00 to 50.2)	0.1	9.96 (1.40 to 70.7)
P for heterogeneity:			*0.01*		*0.02*
Total at least 5 years since diagnosis of DCIS	70977	99	1.39 (1.15 to 1.70)	52.67	1.88 (1.54 to 2.29)
**All years since diagnosis of DCIS**					
Unilateral with surgery	162,820	136	0.84 (0.71 to 0.99)	111.03	1.22 (1.04 to 1.45)
Unilateral with no/unknown surgery	9414	23	2.44 (1.62 to 3.68)	6.75	3.41 (2.27 to 5.13)
Bilateral	349	1	2.87 (0.40 to 20.4)	0.23	4.30 (0.61 to 30.5)
P for heterogeneity:			*<0.001*		*<0.001*
**Total**	172,583	160	0.93 (0.79 to 1.08)	118	1.36 (1.16 to 1.58)

*i. e. estimated ratio of observed rate to expected rate. See supplementary text 1 for further details

Supplementary Table S13. Characteristics and vital status on 31 December 2014 of 24,779 women diagnosed with unilateral DCIS detected as a result of screening between April 2000 and March 2014 and who received surgery. Women recorded with oestrogen-receptor positive DCIS and receiving endocrine therapy are excluded.

	Treatment				
	**BCS + RT**	**BCS - RT**	**Mastectomy**	**P for heterogeneity**	**Total**
**Year of DCIS diagnosis**					
2000-04	531 (10.8)	2734 (55.6)	1651 (33.6)	<0.001	4916 (100.0)
2005-09	1132 (13.2)	4631 (54.0)	2805 (32.7)		8568 (100.0)
2010-14	3136 (27.8)	5076 (44.9)	3083 (27.3)		11,295 (100.0)
**Age at DCIS diagnosis (years)**					
<55	1422 (18.5)	3755 (48.9)	2496 (32.5)	<0.001	7673 (100.0)
55-59	1010 (19.9)	2488 (48.9)	1590 (31.2)		5088 (100.0)
60-64	1054 (19.3)	2802 (51.4)	1592 (29.2)		5448 (100.0)
65+	1312 (20.0)	3396 (51.7)	1860 (28.3)		6568 (100.0)
**Region**					
Eastern	1266 (37.0)	1227 (35.9)	926 (27.1)	<0.001	3419 (100.0)
North West	361 (13.6)	1406 (52.9)	893 (33.6)		2660 (100.0)
Northern/Yorkshire	1510 (39.3)	1026 (26.7)	1310 (34.1)		3846 (100.0)
Oxford	157 (10.4)	937 (62.2)	413 (27.4)		1507 (100.0)
South West	198 (4.9)	2699 (66.6)	1156 (28.5)		4053 (100.0)
Thames	531 (11.2)	2792 (59.0)	1406 (29.7)		4729 (100.0)
Trent	395 (19.4)	942 (46.2)	702 (34.4)		2039 (100.0)
West Midlands	382 (15.1)	1411 (55.9)	731 (29.0)		2524 (100.0)
**DCIS size (mm)**					
<=10	1416 (17.5)	5950 (73.4)	738 (9.1)	<0.001	8104 (100.0)
11-20	1939 (26.7)	3837 (52.7)	1498 (20.6)		7274 (100.0)
21-50	1383 (18.6)	2470 (33.1)	3599 (48.3)		7452 (100.0)
51+	65 (3.3)	185 (9.5)	1704 (87.2)		1954 (100.0)
**DCIS grade**					
Low/intermediate	1202 (12.2)	6647 (67.5)	2003 (20.3)	<0.001	9852 (100.0)
High	3599 (24.1)	5795 (38.8)	5534 (37.1)		14,928 (100.0)
**Oestrogen-receptor status**					
Positive	3579 (19.3)	9948 (53.7)	5000 (27.0)	<0.001	18,527 (100.0)
Negative	1221 (19.5)	2491 (39.9)	2535 (40.6)		6247 (100.0)
**Laterality of DCIS**					
Left	2510 (19.7)	6414 (50.2)	3842 (30.1)	0.36	12,766 (100.0)
Right	2290 (19.1)	6028 (50.2)	3695 (30.8)		12,013 (100.0)
**Final margin distance (mm)**					
5+	2184 (22.6)	4080 (42.2)	3408 (35.2)		9672 (100.0)
3-4	684 (26.0)	1484 (56.3)	466 (17.7)		2634 (100.0)
1-2	855 (23.0)	2094 (56.2)	775 (20.8)		3724 (100.0)
Involved	99 (17.7)	280 (50.2)	179 (32.1)		558 (100.0)
Before 2007	977 (11.9)	4505 (55.0)	2705 (33.0)		8187 (100.0)
**Duration of follow-up (years)**					
0-4	3176 (27.2)	5285 (45.3)	3205 (27.5)		11,666 (100.0)
5-9	1129 (13.2)	4619 (54.0)	2803 (32.8)		8551 (100.0)
10-14	494 (10.8)	2538 (55.6)	1530 (33.5)		4562 (100.0)
**Laterality of IBC**					
Ipsilateral	66 (11.7)	398 (70.6)	100 (17.7)		564 (100.0)
Contralateral	75 (15.2)	238 (48.3)	180 (36.5)		493 (100.0)
Unknown	7 (11.1)	26 (41.3)	30 (47.6)		63 (100.0)
**Vital status on 31 Dec 2014**					
Alive	4673 (19.6)	11,897 (50.0)	7216 (30.3)		23,786 (100.0)
Emigrated	6 (10.5)	32 (56.1)	19 (33.3)		57 (100.0)
Dead	120 (12.8)	512 (54.8)	303 (32.4)		935 (100.0)
**Cause of death**					
Breast cancer	15	49	43		107
Other causes	105	457	249		811
Unknown cause	-	7	10		17
**Total**	4799 (19.4)	12,441 (50.2)	7539 (30.4)		24,779 (100.0)

BCS+RT: breast-conserving surgery, radiotherapy recorded
BCS-RT: breast-conserving surgery, radiotherapy not recorded

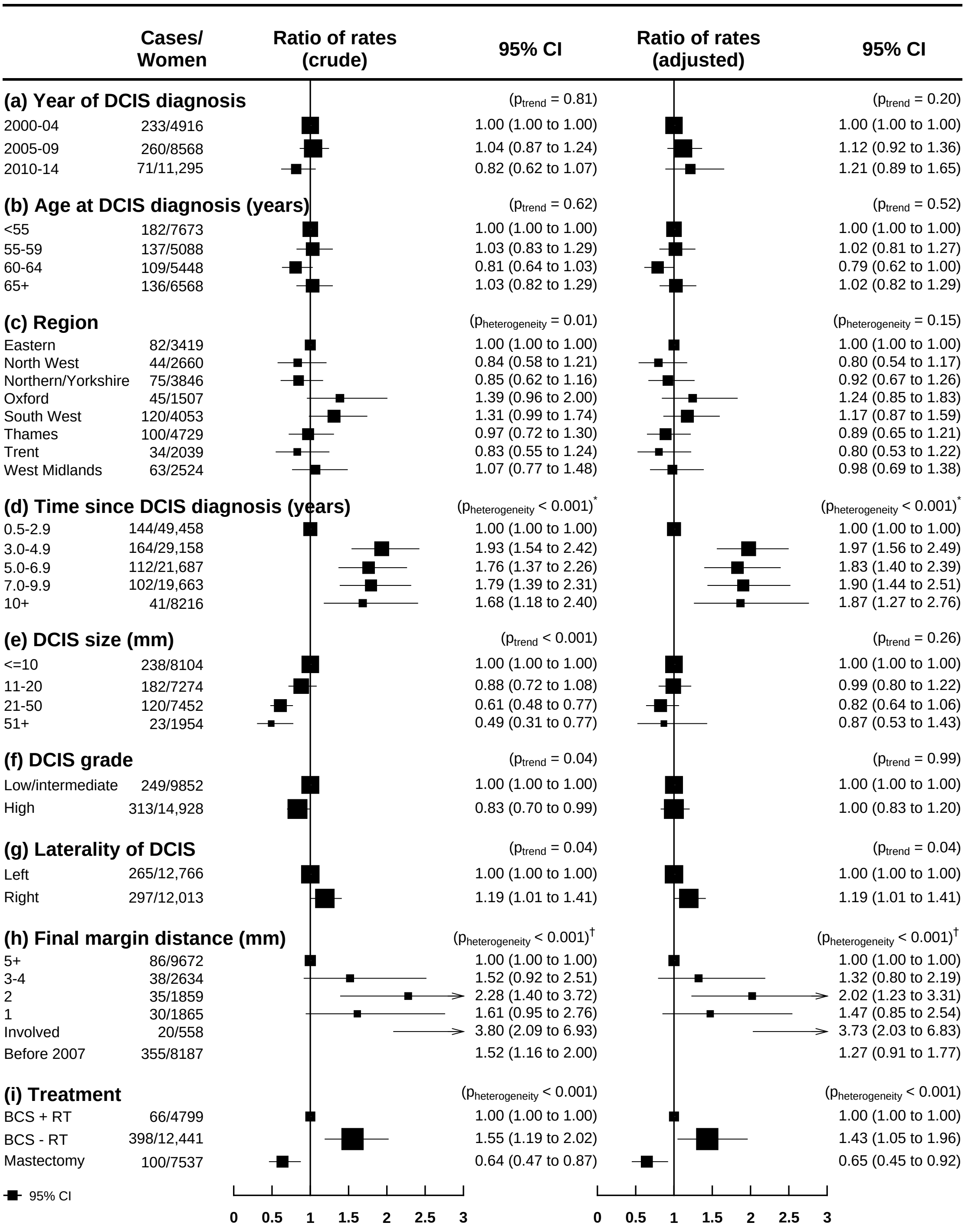

	Cases/ Women	Ratio of rates (crude) 95% CI	Ratio of rates (adjusted) 95% CI
**(a) Year of DCIS diagnosis**		(p_{trend} = 0.81)	(p_{trend} = 0.20)
2000-04	233/4916	1.00 (1.00 to 1.00)	1.00 (1.00 to 1.00)
2005-09	260/8568	1.04 (0.87 to 1.24)	1.12 (0.92 to 1.36)
2010-14	71/11,295	0.82 (0.62 to 1.07)	1.21 (0.89 to 1.65)
**(b) Age at DCIS diagnosis (years)**		(p_{trend} = 0.62)	(p_{trend} = 0.52)
<55	182/7673	1.00 (1.00 to 1.00)	1.00 (1.00 to 1.00)
55-59	137/5088	1.03 (0.83 to 1.29)	1.02 (0.81 to 1.27)
60-64	109/5448	0.81 (0.64 to 1.03)	0.79 (0.62 to 1.00)
65+	136/6568	1.03 (0.82 to 1.29)	1.02 (0.82 to 1.29)
**(c) Region**		($p_{heterogeneity}$ = 0.01)	($p_{heterogeneity}$ = 0.15)
Eastern	82/3419	1.00 (1.00 to 1.00)	1.00 (1.00 to 1.00)
North West	44/2660	0.84 (0.58 to 1.21)	0.80 (0.54 to 1.17)
Northern/Yorkshire	75/3846	0.85 (0.62 to 1.16)	0.92 (0.67 to 1.26)
Oxford	45/1507	1.39 (0.96 to 2.00)	1.24 (0.85 to 1.83)
South West	120/4053	1.31 (0.99 to 1.74)	1.17 (0.87 to 1.59)
Thames	100/4729	0.97 (0.72 to 1.30)	0.89 (0.65 to 1.21)
Trent	34/2039	0.83 (0.55 to 1.24)	0.80 (0.53 to 1.22)
West Midlands	63/2524	1.07 (0.77 to 1.48)	0.98 (0.69 to 1.38)
**(d) Time since DCIS diagnosis (years)**		($p_{heterogeneity}$ < 0.001)*	($p_{heterogeneity}$ < 0.001)*
0.5-2.9	144/49,458	1.00 (1.00 to 1.00)	1.00 (1.00 to 1.00)
3.0-4.9	164/29,158	1.93 (1.54 to 2.42)	1.97 (1.56 to 2.49)
5.0-6.9	112/21,687	1.76 (1.37 to 2.26)	1.83 (1.40 to 2.39)
7.0-9.9	102/19,663	1.79 (1.39 to 2.31)	1.90 (1.44 to 2.51)
10+	41/8216	1.68 (1.18 to 2.40)	1.87 (1.27 to 2.76)
**(e) DCIS size (mm)**		(p_{trend} < 0.001)	(p_{trend} = 0.26)
<=10	238/8104	1.00 (1.00 to 1.00)	1.00 (1.00 to 1.00)
11-20	182/7274	0.88 (0.72 to 1.08)	0.99 (0.80 to 1.22)
21-50	120/7452	0.61 (0.48 to 0.77)	0.82 (0.64 to 1.06)
51+	23/1954	0.49 (0.31 to 0.77)	0.87 (0.53 to 1.43)
**(f) DCIS grade**		(p_{trend} = 0.04)	(p_{trend} = 0.99)
Low/intermediate	249/9852	1.00 (1.00 to 1.00)	1.00 (1.00 to 1.00)
High	313/14,928	0.83 (0.70 to 0.99)	1.00 (0.83 to 1.20)
**(g) Laterality of DCIS**		(p_{trend} = 0.04)	(p_{trend} = 0.04)
Left	265/12,766	1.00 (1.00 to 1.00)	1.00 (1.00 to 1.00)
Right	297/12,013	1.19 (1.01 to 1.41)	1.19 (1.01 to 1.41)
**(h) Final margin distance (mm)**		($p_{heterogeneity}$ < 0.001)†	($p_{heterogeneity}$ < 0.001)†
5+	86/9672	1.00 (1.00 to 1.00)	1.00 (1.00 to 1.00)
3-4	38/2634	1.52 (0.92 to 2.51)	1.32 (0.80 to 2.19)
2	35/1859	2.28 (1.40 to 3.72)	2.02 (1.23 to 3.31)
1	30/1865	1.61 (0.95 to 2.76)	1.47 (0.85 to 2.54)
Involved	20/558	3.80 (2.09 to 6.93)	3.73 (2.03 to 6.83)
Before 2007	355/8187	1.52 (1.16 to 2.00)	1.27 (0.91 to 1.77)
**(i) Treatment**		($p_{heterogeneity}$ < 0.001)	($p_{heterogeneity}$ < 0.001)
BCS + RT	66/4799	1.00 (1.00 to 1.00)	1.00 (1.00 to 1.00)
BCS - RT	398/12,441	1.55 (1.19 to 2.02)	1.43 (1.05 to 1.96)
Mastectomy	100/7537	0.64 (0.47 to 0.87)	0.65 (0.45 to 0.92)

■ 95% CI

BCS+RT: breast-conserving surgery, radiotherapy recorded
BCS-RT: breast-conserving surgery, radiotherapy not recorded
CI: confidence interval
P: results of tests for heterogeneity or trend
* Tests for trend excluding years 0.5-2.9: crude P=0.40; adjusted P=0.87
† Tests for trend across clear margin categories: crude P=0.004; adjusted P=0.02

Supplementary Figure S1. Incidence of ipsilateral invasive breast cancer according to various factors in 24,779 women diagnosed with unilateral DCIS as a result of screening between April 2000 and March 2014 and who received surgery showing final margin distances of 1 and 2 mm separately. Women recorded with oestrogen-receptor positive DCIS and receiving endocrine therapy are excluded. For each factor rates are shown relative to first category and adjustment is for all other factors except final margin distance. Final margin distance was not included in the adjustment as information on this variable was available only from 2007 onwards.

Breast-conserving surgery with radiotherapy

	Cases/ Women	Ratio of rates (crude) 95% CI	Ratio of rates (adjusted) 95% CI
**(a) Year of DCIS diagnosis**		(p_{trend} = 0.19)	(p_{trend} = 0.93)
2000-04	26/531	1.00 (1.00 to 1.00)	1.00 (1.00 to 1.00)
2005-09	27/1132	0.77 (0.45 to 1.34)	0.92 (0.51 to 1.67)
2010-14	13/3136	0.62 (0.32 to 1.21)	1.33 (0.52 to 3.41)
**(b) Age at DCIS diagnosis (years)**		(p_{trend} = 0.20)	(p_{trend} = 0.17)
<55	14/1422	1.00 (1.00 to 1.00)	1.00 (1.00 to 1.00)
55-59	25/1010	2.40 (1.23 to 4.69)	2.44 (1.24 to 4.78)
60-64	16/1054	1.53 (0.74 to 3.18)	1.56 (0.75 to 3.26)
65+	12/1312	1.04 (0.47 to 2.31)	1.08 (0.48 to 2.39)
**(c) Region**		($p_{heterogeneity}$ = 0.63)	($p_{heterogeneity}$ = 0.27)
Eastern	28/1266	1.00 (1.00 to 1.00)	1.00 (1.00 to 1.00)
North West	3/361	1.85 (0.56 to 6.08)	3.74 (0.94 to 14.98)
Northern/York.	29/1510	0.93 (0.55 to 1.57)	0.90 (0.53 to 1.53)
Oxford	0/157		
South West	0/198		
Thames	1/531	0.21 (0.03 to 1.54)	0.22 (0.03 to 1.64)
Trent	4/395	1.56 (0.55 to 4.44)	2.56 (0.83 to 7.92)
West Midlands	1/382	0.42 (0.06 to 3.06)	0.70 (0.09 to 5.68)
**(d) Time since DCIS diagnosis (years)**		($p_{heterogeneity}$ = 0.002)*	($p_{heterogeneity}$ = 0.001)*
0.5-2.9	12/49458	1.00 (1.00 to 1.00)	1.00 (1.00 to 1.00)
3.0-4.9	23/29158	4.03 (2.00 to 8.12)	5.33 (2.44 to 11.66)
5.0-6.9	13/21687	3.27 (1.49 to 7.17)	4.78 (1.88 to 12.15)
7.0-9.9	13/19663	3.92 (1.79 to 8.57)	5.63 (2.18 to 14.50)
10+	5/8216	3.75 (1.23 to 11.42)	5.38 (1.48 to 19.49)
**(e) DCIS size (mm)**		(p_{trend} = 0.55)	(p_{trend} = 0.45)
<=10	19/1416	1.00 (1.00 to 1.00)	1.00 (1.00 to 1.00)
11-20	27/1939	1.10 (0.59 to 2.07)	1.07 (0.57 to 2.02)
21-50	20/1383	1.38 (0.71 to 2.69)	1.44 (0.73 to 2.85)
51+	0/65		
**(f) DCIS grade**		(p_{trend} = 0.73)	(p_{trend} = 0.62)
Low/inter.	18/1202	1.00 (1.00 to 1.00)	1.00 (1.00 to 1.00)
High	47/3599	0.91 (0.51 to 1.60)	0.86 (0.49 to 1.53)
**(g) Laterality of DCIS**		(p_{trend} = 0.42)	(p_{trend} = 0.49)
Left	38/2510	1.00 (1.00 to 1.00)	1.00 (1.00 to 1.00)
Right	28/2290	0.82 (0.50 to 1.34)	0.84 (0.51 to 1.38)
**(h) Final margin distance (mm)**		($p_{heterogeneity}$ = 0.47)†	($p_{heterogeneity}$ = 0.51)†
5+	9/2184	1.00 (1.00 to 1.00)	1.00 (1.00 to 1.00)
3-4	5/684	1.62 (0.41 to 6.48)	1.58 (0.39 to 6.38)
1-2	9/855	2.40 (0.77 to 7.51)	2.33 (0.73 to 7.42)
Involved	1/99		
Before 2007	43/977	2.59 (1.13 to 5.96)	2.62 (0.91 to 7.56)

■ 95% CI (axes: 0, 0.5, 1, 1.5, 2, 2.5, 3)

* Tests for trend excluding years 0.5-2.9: crude P=0.90; adjusted P=0.97
† Tests for trend across clear margin categories: crude P=0.13; adjusted P=0.13

Breast-conserving surgery no radiotherapy

	Cases/ Women	Ratio of rates (crude) 95% CI	Ratio of rates (adjusted) 95% CI
**(a) Year of DCIS diagnosis**		(p_{trend} = 0.88)	(p_{trend} = 0.33)
2000-04	170/2734	1.00 (1.00 to 1.00)	1.00 (1.00 to 1.00)
2005-09	179/4631	1.01 (0.82 to 1.25)	1.10 (0.87 to 1.39)
2010-14	49/5076	0.91 (0.66 to 1.25)	1.27 (0.88 to 1.83)
**(b) Age at DCIS diagnosis (years)**		(p_{trend} = 0.92)	(p_{trend} = 0.95)
<55	125/3755	1.00 (1.00 to 1.00)	1.00 (1.00 to 1.00)
55-59	89/2488	0.98 (0.74 to 1.28)	0.98 (0.74 to 1.28)
60-64	77/2802	0.80 (0.60 to 1.07)	0.80 (0.60 to 1.06)
65+	106/3396	1.15 (0.89 to 1.49)	1.17 (0.90 to 1.53)
**(c) Region**		($p_{heterogeneity}$ = 0.47)	($p_{heterogeneity}$ = 0.46)
Eastern	44/1227	1.00 (1.00 to 1.00)	1.00 (1.00 to 1.00)
North West	32/1406	0.67 (0.42 to 1.05)	0.69 (0.44 to 1.10)
Northern/York.	27/1026	0.77 (0.48 to 1.25)	0.79 (0.49 to 1.28)
Oxford	37/937	1.08 (0.69 to 1.67)	1.14 (0.73 to 1.79)
South West	93/2699	0.96 (0.67 to 1.39)	1.01 (0.70 to 1.45)
Thames	85/2792	0.85 (0.59 to 1.23)	0.89 (0.61 to 1.29)
Trent	27/942	0.75 (0.46 to 1.22)	0.79 (0.48 to 1.28)
West Midlands	52/1411	0.90 (0.60 to 1.35)	0.94 (0.63 to 1.42)
**(d) Time since DCIS diagnosis (years)**		($p_{heterogeneity}$ < 0.001)*	($p_{heterogeneity}$ < 0.001)*
0.5-2.9	104/49458	1.00 (1.00 to 1.00)	1.00 (1.00 to 1.00)
3.0-4.9	112/29158	1.76 (1.35 to 2.30)	1.83 (1.39 to 2.42)
5.0-6.9	74/21687	1.54 (1.14 to 2.08)	1.65 (1.19 to 2.27)
7.0-9.9	73/19663	1.68 (1.24 to 2.26)	1.84 (1.32 to 2.55)
10+	34/8216	1.84 (1.24 to 2.72)	2.11 (1.37 to 3.25)
**(e) DCIS size (mm)**		(p_{trend} = 0.15)	(p_{trend} = 0.18)
<=10	203/5950	1.00 (1.00 to 1.00)	1.00 (1.00 to 1.00)
11-20	129/3837	0.99 (0.78 to 1.24)	1.00 (0.79 to 1.26)
21-50	61/2470	0.79 (0.59 to 1.07)	0.80 (0.59 to 1.09)
51+	4/185	0.67 (0.22 to 2.05)	0.65 (0.21 to 2.01)
**(f) DCIS grade**		(p_{trend} = 0.68)	(p_{trend} = 0.89)
Low/inter.	206/6647	1.00 (1.00 to 1.00)	1.00 (1.00 to 1.00)
High	191/5795	0.96 (0.78 to 1.17)	0.98 (0.79 to 1.22)
**(g) Laterality of DCIS**		(p_{trend} = 0.08)	(p_{trend} = 0.07)
Left	187/6414	1.00 (1.00 to 1.00)	1.00 (1.00 to 1.00)
Right	210/6028	1.19 (0.98 to 1.46)	1.20 (0.98 to 1.46)
**(h) Final margin distance (mm)**		($p_{heterogeneity}$ < 0.001)†	($p_{heterogeneity}$ < 0.001)†
5+	56/4080	1.00 (1.00 to 1.00)	1.00 (1.00 to 1.00)
3-4	28/1484	1.26 (0.70 to 2.30)	1.27 (0.70 to 2.31)
1-2	47/2094	1.61 (1.00 to 2.61)	1.68 (1.03 to 2.74)
Involved	16/280	4.04 (2.01 to 8.08)	4.32 (2.13 to 8.78)
Before 2007	250/4505	1.34 (0.95 to 1.89)	1.17 (0.78 to 1.77)

■ 95% CI (axes: 0, 0.5, 1, 1.5, 2, 2.5, 3)

* Tests for trend excluding years 0.5-2.9: crude P=0.98; adjusted P=0.61
† Tests for trend across clear margin categories: crude P=0.05; adjusted P=0.05

Mastectomy

	Cases/ Women	Ratio of rates (crude) 95% CI	Ratio of rates (adjusted) 95% CI
**(a) Year of DCIS diagnosis**		(p_{trend} = 0.31)	(p_{trend} = 0.30)
2000-04	37/1651	1.00 (1.00 to 1.00)	1.00 (1.00 to 1.00)
2005-09	54/2805	1.39 (0.91 to 2.13)	1.33 (0.85 to 2.08)
2010-14	9/3083	0.74 (0.36 to 1.54)	0.89 (0.40 to 1.97)
**(b) Age at DCIS diagnosis (years)**		(p_{trend} = 0.02)	(p_{trend} = 0.01)
<55	43/2496	1.00 (1.00 to 1.00)	1.00 (1.00 to 1.00)
55-59	23/1590	0.74 (0.44 to 1.23)	0.71 (0.42 to 1.18)
60-64	16/1592	0.53 (0.30 to 0.96)	0.52 (0.29 to 0.93)
65+	18/1860	0.63 (0.36 to 1.09)	0.58 (0.33 to 1.01)
**(c) Region**		($p_{heterogeneity}$ = 0.05)	($p_{heterogeneity}$ = 0.05)
Eastern	10/926	1.00 (1.00 to 1.00)	1.00 (1.00 to 1.00)
North West	9/893	1.09 (0.44 to 2.74)	1.11 (0.44 to 2.77)
Northern/York.	19/1310	1.39 (0.63 to 3.04)	1.40 (0.64 to 3.07)
Oxford	8/413	2.04 (0.79 to 5.24)	2.06 (0.80 to 5.32)
South West	27/1156	2.31 (1.09 to 4.87)	2.32 (1.10 to 4.91)
Thames	14/1406	1.02 (0.45 to 2.35)	1.00 (0.44 to 2.30)
Trent	3/702	0.49 (0.13 to 1.81)	0.49 (0.13 to 1.80)
West Midlands	10/731	1.27 (0.52 to 3.11)	1.31 (0.53 to 3.20)
**(d) Time since DCIS diagnosis (years)**		($p_{heterogeneity}$ = 0.05)*	($p_{heterogeneity}$ = 0.12)*
0.5-2.9	28/49458	1.00 (1.00 to 1.00)	1.00 (1.00 to 1.00)
3.0-4.9	29/29158	1.70 (1.01 to 2.85)	1.61 (0.95 to 2.74)
5.0-6.9	25/21687	1.91 (1.11 to 3.28)	1.76 (0.99 to 3.11)
7.0-9.9	16/19663	1.37 (0.74 to 2.52)	1.31 (0.69 to 2.52)
10+	2/8216	0.34 (0.06 to 1.90)	0.35 (0.06 to 2.02)
**(e) DCIS size (mm)**		(p_{trend} = 0.61)	(p_{trend} = 0.53)
<=10	16/738	1.00 (1.00 to 1.00)	1.00 (1.00 to 1.00)
11-20	26/1498	0.88 (0.45 to 1.70)	0.91 (0.47 to 1.78)
21-50	39/3599	0.64 (0.35 to 1.18)	0.64 (0.35 to 1.19)
51+	19/1704	0.80 (0.40 to 1.63)	0.78 (0.38 to 1.61)
**(f) DCIS grade**		(p_{trend} = 0.53)	(p_{trend} = 0.38)
Low/inter.	25/2003	1.00 (1.00 to 1.00)	1.00 (1.00 to 1.00)
High	75/5534	1.17 (0.72 to 1.88)	1.24 (0.77 to 2.01)
**(g) Laterality of DCIS**		(p_{trend} = 0.04)	(p_{trend} = 0.03)
Left	40/3842	1.00 (1.00 to 1.00)	1.00 (1.00 to 1.00)
Right	59/3695	1.53 (1.02 to 2.28)	1.54 (1.03 to 2.31)
**(h) Final margin distance (mm)**		($p_{heterogeneity}$ = 0.54)†	($p_{heterogeneity}$ = 0.66)†
5+	21/3408	1.00 (1.00 to 1.00)	1.00 (1.00 to 1.00)
3-4	5/466	1.53 (0.47 to 5.04)	1.45 (0.44 to 4.78)
1-2	9/775	1.81 (0.68 to 4.80)	1.65 (0.62 to 4.39)
Involved	3/179	2.28 (0.41 to 12.65)	2.12 (0.38 to 11.91)
Before 2007	62/2705	1.26 (0.73 to 2.15)	1.18 (0.61 to 2.27)

■ 95% CI (axes: 0, 0.5, 1, 1.5, 2, 2.5, 3)

* Tests for trend excluding years 0.5-2.9: crude P=0.06; adjusted P=0.19
† Tests for trend across clear margin categories: crude P=0.20; adjusted P=0.31

Supplementary Figure S2. Incidence of ipsilateral invasive breast cancer according to various factors in 24,779 women diagnosed with unilateral DCIS detected as a result of screening between April 2000 and March 2014 and who received surgery. Women with oestrogen-receptor positive DCIS and recorded as receiving endocrine therapy are excluded. For each factor rates are shown relative to first category and adjustment is for all other factors except final margin distance. Final margin distance was not included in the adjustment as information on this variable was available only from 2007 onwards.

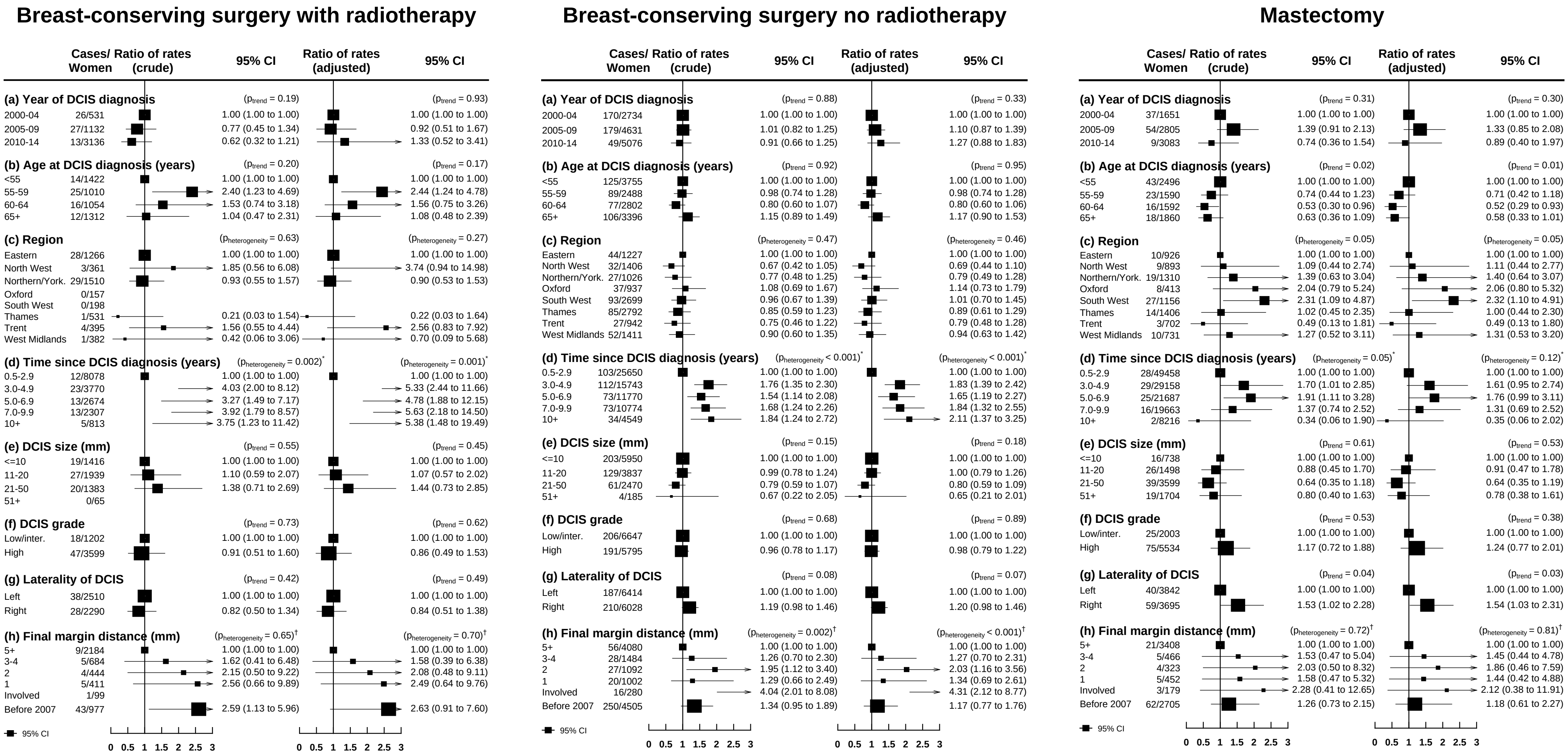

Breast-conserving surgery with radiotherapy:
* Tests for trend excluding years 0.5-2.9: crude P=0.90; adjusted P=0.97
† Tests for trend across clear margin categories: crude P=0.12; adjusted P=0.12

Breast-conserving surgery no radiotherapy:
* Tests for trend excluding years 0.5-2.9: crude P=0.98; adjusted P=0.61
† Tests for trend across clear margin categories: crude P=0.11; adjusted P=0.10

Mastectomy:
* Tests for trend excluding years 0.5-2.9: crude P=0.06; adjusted P=0.19
† Tests for trend across clear margin categories: crude P=0.24; adjusted P=0.37

Supplementary Figure S3. Incidence of ipsilateral invasive breast cancer according to various factors in 24,779 women diagnosed with unilateral DCIS detected as a result of screening between April 2000 and March 2014 and who received surgery showing final margin distances of 1 and 2 mm separately. Women with oestrogen-receptor positive DCIS and recorded as receiving endocrine therapy are excluded. For each factor rates are shown relative to first category and adjustment is for all other factors except final margin distance. Final margin distance was not included in the adjustment as information on this variable was available only from 2007 onwards.

Supplementary Table S14. Tests for interactions between pairs of factors for analysis shown in Figure 3.

Factors	P-value
Treatment x age at diagnosis	0.02
Treatment x year of diagnosis	0.36
Treatment x time since diagnosis	0.16
Treatment x DCIS grade	0.72
Treatment x tumour size	0.46
Treatment x laterality	0.15
DCIS grade x age at diagnosis	0.18
DCIS grade x year of diagnosis	0.19
DCIS grade x time since diagnosis	0.01
DCIS grade x tumour size	0.90
DCIS grade x laterality	0.46
Tumour size x age at diagnosis	0.47
Tumour size x year of diagnosis	0.82
Tumour size x time since diagnosis	0.26
Tumour size x laterality	0.26
Age at diagnosis x year of diagnosis	0.39
Age at diagnosis x time since diagnosis	0.58
Age at diagnosis x laterality	0.20
Year of diagnosis x time since diagnosis	0.77
Year of diagnosis x laterality	0.07
Time since diagnosis x laterality	0.70
*Final margin distance x treatment	0.98
Final margin distance x DCIS grade	0.43
Final margin distance x age at diagnosis	0.85
Final margin distance x year of diagnosis	0.59
Final margin distance x time since diagnosis	0.54
Final margin distance x tumour size	0.80
Final margin distance x laterality	0.50

* Women diagnosed before 2007 were excluded for testing interaction between final marginal distance and other factors.

Factors are treated as categorical variables with categories as shown in Figure 3, apart from tumour size and final margin distance which are treated as continuous variables in this table. Adjustments are as in Figure 3 except for analyses including interactions with final margin distance, in which final margin distance is included as a main effect as well as an interaction.

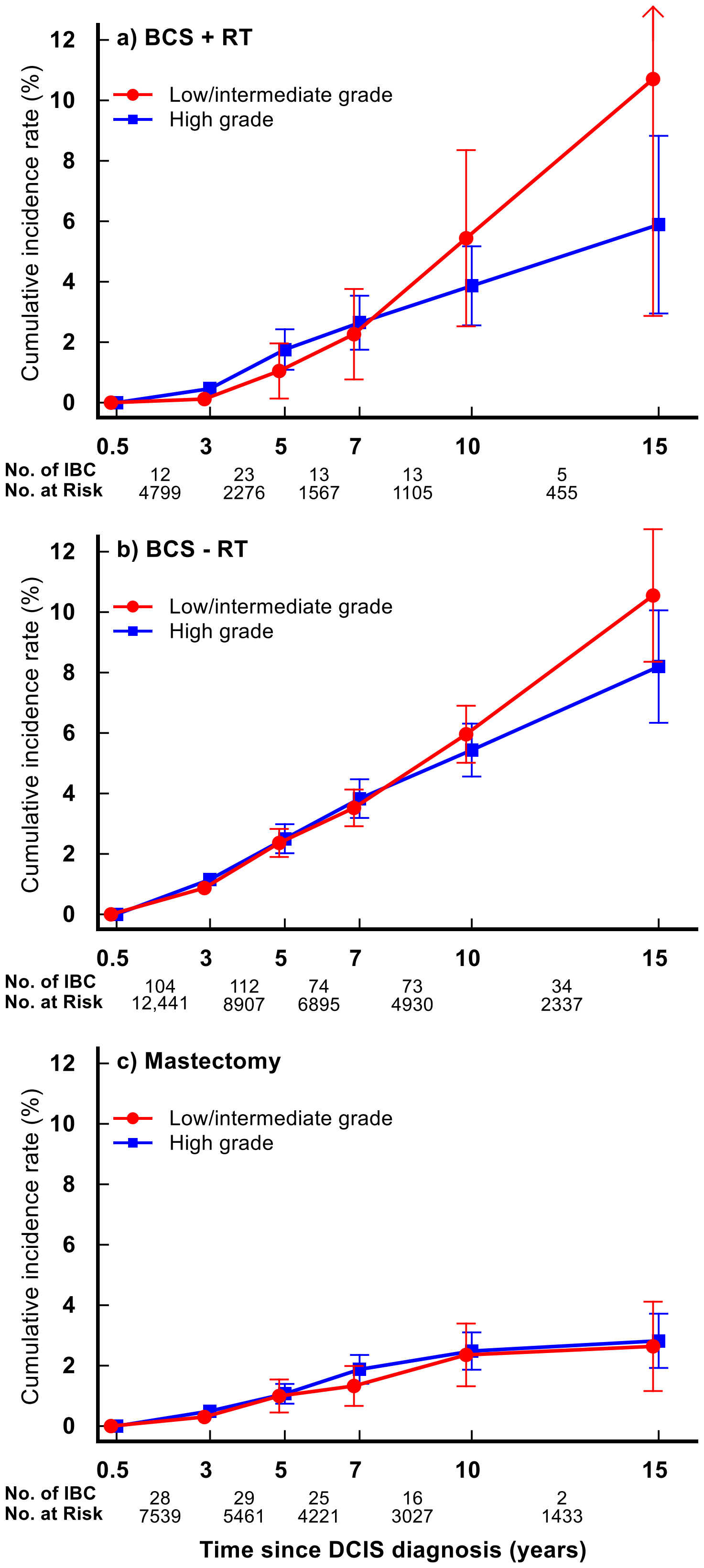

BCS+RT: breast-conserving surgery, radiotherapy recorded
BCS-RT: breast-conserving surgery, radiotherapy not recorded
No. of IBC: Number of ipsilateral invasive breast cancers during interval
No. at Risk: Number of individuals at risk of invasive breast cancer at start of interval

Supplementary Figure S4. Cumulative incidence rate of ipsilateral invasive breast cancer in 24,779 women diagnosed with unilateral DCIS detected as a result of screening between April 2000 and March 2014 by treatment and DCIS grade. Women with no surgery and women with oestrogen-receptor positive DCIS and recorded as receiving endocrine therapy were excluded.

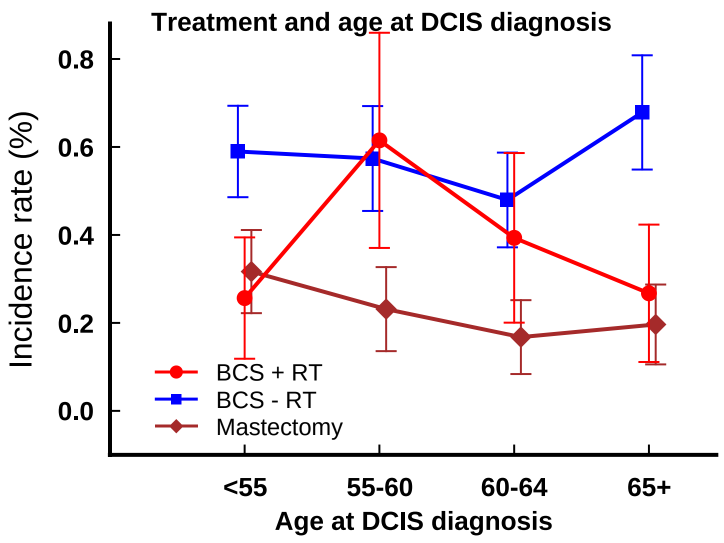

BCS+RT: breast-conserving surgery, radiotherapy recorded
BCS-RT: breast-conserving surgery, radiotherapy not recorded

Supplementary Figure S5. Annual incidence rate of ipsilateral invasive breast cancer in 24,779 women diagnosed with unilateral DCIS detected as a result of screening between April 2000 and March 2014 and who received surgery by treatment and age at diagnosis of DCIS. Women with oestrogen-receptor positive DCIS and recorded as receiving endocrine therapy are excluded.

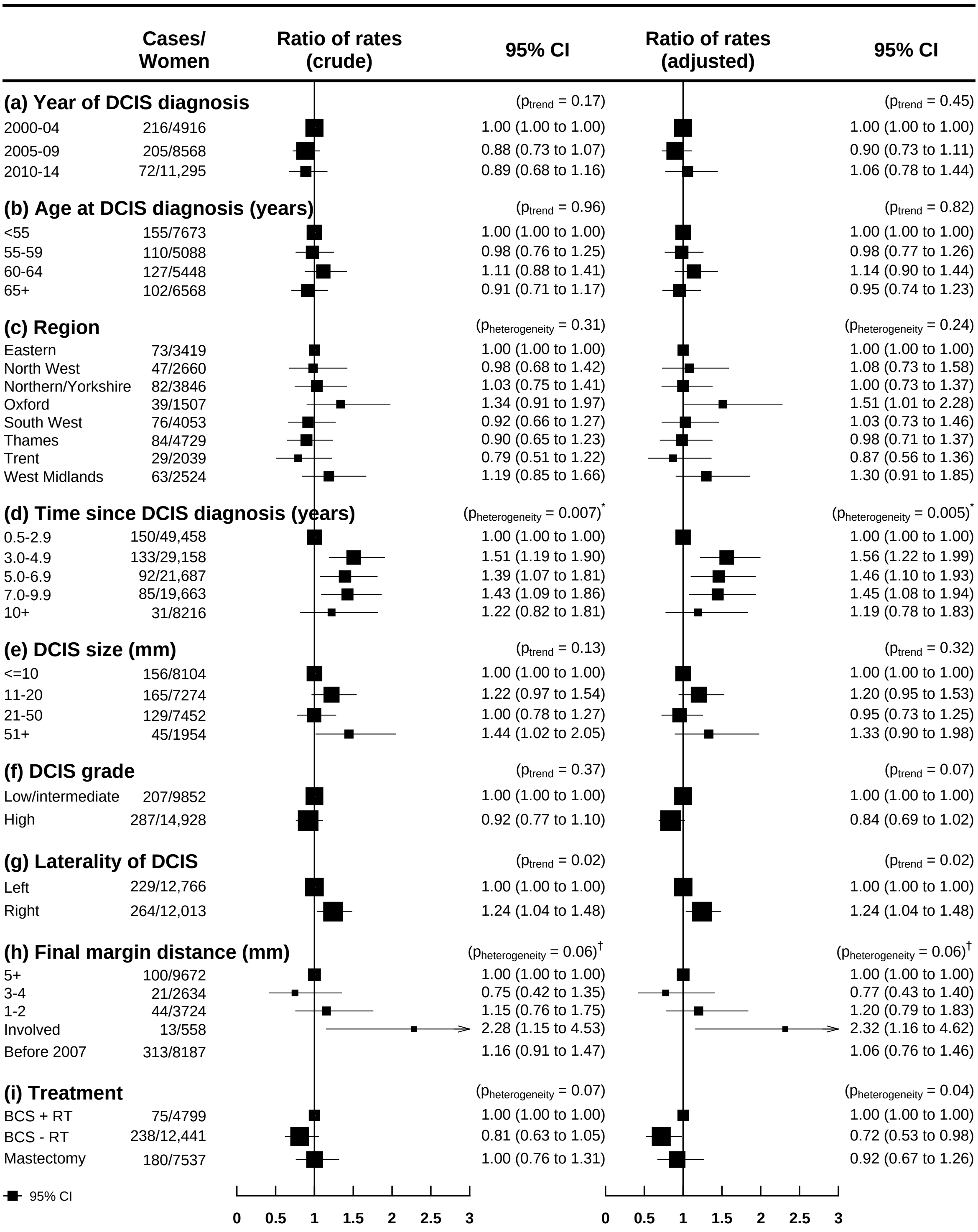

	Cases/ Women	Ratio of rates (crude) 95% CI	Ratio of rates (adjusted) 95% CI
**(a) Year of DCIS diagnosis**		(p_{trend} = 0.17)	(p_{trend} = 0.45)
2000-04	216/4916	1.00 (1.00 to 1.00)	1.00 (1.00 to 1.00)
2005-09	205/8568	0.88 (0.73 to 1.07)	0.90 (0.73 to 1.11)
2010-14	72/11,295	0.89 (0.68 to 1.16)	1.06 (0.78 to 1.44)
**(b) Age at DCIS diagnosis (years)**		(p_{trend} = 0.96)	(p_{trend} = 0.82)
<55	155/7673	1.00 (1.00 to 1.00)	1.00 (1.00 to 1.00)
55-59	110/5088	0.98 (0.76 to 1.25)	0.98 (0.77 to 1.26)
60-64	127/5448	1.11 (0.88 to 1.41)	1.14 (0.90 to 1.44)
65+	102/6568	0.91 (0.71 to 1.17)	0.95 (0.74 to 1.23)
**(c) Region**		($p_{heterogeneity}$ = 0.31)	($p_{heterogeneity}$ = 0.24)
Eastern	73/3419	1.00 (1.00 to 1.00)	1.00 (1.00 to 1.00)
North West	47/2660	0.98 (0.68 to 1.42)	1.08 (0.73 to 1.58)
Northern/Yorkshire	82/3846	1.03 (0.75 to 1.41)	1.00 (0.73 to 1.37)
Oxford	39/1507	1.34 (0.91 to 1.97)	1.51 (1.01 to 2.28)
South West	76/4053	0.92 (0.66 to 1.27)	1.03 (0.73 to 1.46)
Thames	84/4729	0.90 (0.65 to 1.23)	0.98 (0.71 to 1.37)
Trent	29/2039	0.79 (0.51 to 1.22)	0.87 (0.56 to 1.36)
West Midlands	63/2524	1.19 (0.85 to 1.66)	1.30 (0.91 to 1.85)
**(d) Time since DCIS diagnosis (years)**		($p_{heterogeneity}$ = 0.007)*	($p_{heterogeneity}$ = 0.005)*
0.5-2.9	150/49,458	1.00 (1.00 to 1.00)	1.00 (1.00 to 1.00)
3.0-4.9	133/29,158	1.51 (1.19 to 1.90)	1.56 (1.22 to 1.99)
5.0-6.9	92/21,687	1.39 (1.07 to 1.81)	1.46 (1.10 to 1.93)
7.0-9.9	85/19,663	1.43 (1.09 to 1.86)	1.45 (1.08 to 1.94)
10+	31/8216	1.22 (0.82 to 1.81)	1.19 (0.78 to 1.83)
**(e) DCIS size (mm)**		(p_{trend} = 0.13)	(p_{trend} = 0.32)
<=10	156/8104	1.00 (1.00 to 1.00)	1.00 (1.00 to 1.00)
11-20	165/7274	1.22 (0.97 to 1.54)	1.20 (0.95 to 1.53)
21-50	129/7452	1.00 (0.78 to 1.27)	0.95 (0.73 to 1.25)
51+	45/1954	1.44 (1.02 to 2.05)	1.33 (0.90 to 1.98)
**(f) DCIS grade**		(p_{trend} = 0.37)	(p_{trend} = 0.07)
Low/intermediate	207/9852	1.00 (1.00 to 1.00)	1.00 (1.00 to 1.00)
High	287/14,928	0.92 (0.77 to 1.10)	0.84 (0.69 to 1.02)
**(g) Laterality of DCIS**		(p_{trend} = 0.02)	(p_{trend} = 0.02)
Left	229/12,766	1.00 (1.00 to 1.00)	1.00 (1.00 to 1.00)
Right	264/12,013	1.24 (1.04 to 1.48)	1.24 (1.04 to 1.48)
**(h) Final margin distance (mm)**		($p_{heterogeneity}$ = 0.06)†	($p_{heterogeneity}$ = 0.06)†
5+	100/9672	1.00 (1.00 to 1.00)	1.00 (1.00 to 1.00)
3-4	21/2634	0.75 (0.42 to 1.35)	0.77 (0.43 to 1.40)
1-2	44/3724	1.15 (0.76 to 1.75)	1.20 (0.79 to 1.83)
Involved	13/558	2.28 (1.15 to 4.53)	2.32 (1.16 to 4.62)
Before 2007	313/8187	1.16 (0.91 to 1.47)	1.06 (0.76 to 1.46)
**(i) Treatment**		($p_{heterogeneity}$ = 0.07)	($p_{heterogeneity}$ = 0.04)
BCS + RT	75/4799	1.00 (1.00 to 1.00)	1.00 (1.00 to 1.00)
BCS - RT	238/12,441	0.81 (0.63 to 1.05)	0.72 (0.53 to 0.98)
Mastectomy	180/7537	1.00 (0.76 to 1.31)	0.92 (0.67 to 1.26)

BCS+RT: breast-conserving surgery, radiotherapy recorded
BCS-RT: breast-conserving surgery, radiotherapy not recorded
CI: confidence interval
P: results of tests for heterogeneity or trend
* Tests for trend excluding years 0.5-2.9: crude P=0.36; adjusted P=0.36
† Tests for trend across clear margin categories: crude P=0.66; adjusted P=0.53

Supplementary Figure S6. Incidence rate of contralateral invasive breast cancer according to various factors in 24,779 women diagnosed with unilateral DCIS detected as a result of screening between April 2000 and March 2014 and who received surgery. Women with oestrogen-receptor positive DCIS and recorded as receiving endocrine therapy are excluded. For each factor, rates are shown relative to the first category and adjustment is for all other factors except final margin distance. Final margin distance was not included in the adjustment as information on this variable was available only from 2007 onwards.

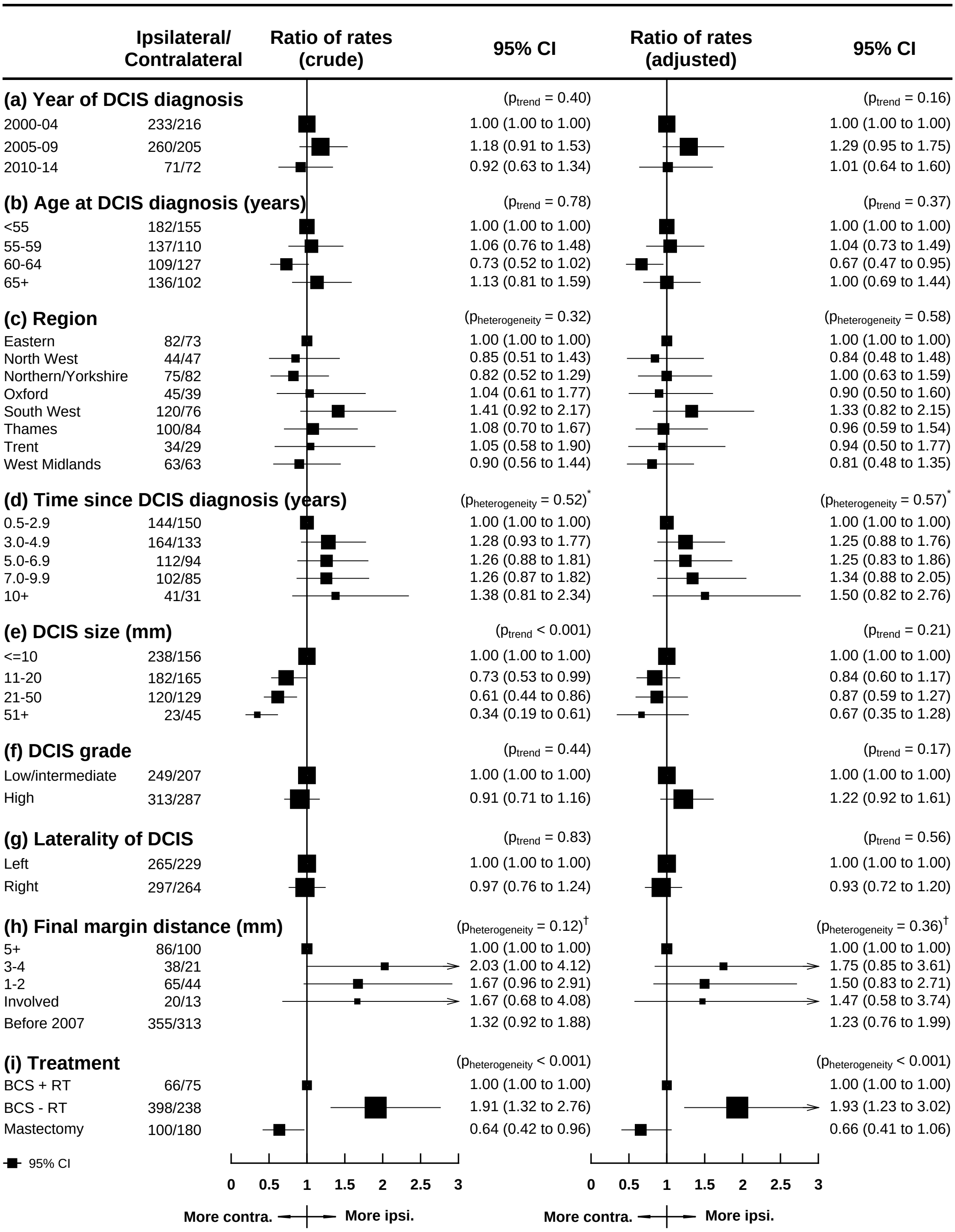

	Ipsilateral/ Contralateral	Ratio of rates (crude) 95% CI	Ratio of rates (adjusted) 95% CI
**(a) Year of DCIS diagnosis**		(p_{trend} = 0.40)	(p_{trend} = 0.16)
2000-04	233/216	1.00 (1.00 to 1.00)	1.00 (1.00 to 1.00)
2005-09	260/205	1.18 (0.91 to 1.53)	1.29 (0.95 to 1.75)
2010-14	71/72	0.92 (0.63 to 1.34)	1.01 (0.64 to 1.60)
**(b) Age at DCIS diagnosis (years)**		(p_{trend} = 0.78)	(p_{trend} = 0.37)
<55	182/155	1.00 (1.00 to 1.00)	1.00 (1.00 to 1.00)
55-59	137/110	1.06 (0.76 to 1.48)	1.04 (0.73 to 1.49)
60-64	109/127	0.73 (0.52 to 1.02)	0.67 (0.47 to 0.95)
65+	136/102	1.13 (0.81 to 1.59)	1.00 (0.69 to 1.44)
**(c) Region**		($p_{heterogeneity}$ = 0.32)	($p_{heterogeneity}$ = 0.58)
Eastern	82/73	1.00 (1.00 to 1.00)	1.00 (1.00 to 1.00)
North West	44/47	0.85 (0.51 to 1.43)	0.84 (0.48 to 1.48)
Northern/Yorkshire	75/82	0.82 (0.52 to 1.29)	1.00 (0.63 to 1.59)
Oxford	45/39	1.04 (0.61 to 1.77)	0.90 (0.50 to 1.60)
South West	120/76	1.41 (0.92 to 2.17)	1.33 (0.82 to 2.15)
Thames	100/84	1.08 (0.70 to 1.67)	0.96 (0.59 to 1.54)
Trent	34/29	1.05 (0.58 to 1.90)	0.94 (0.50 to 1.77)
West Midlands	63/63	0.90 (0.56 to 1.44)	0.81 (0.48 to 1.35)
**(d) Time since DCIS diagnosis (years)**		($p_{heterogeneity}$ = 0.52)*	($p_{heterogeneity}$ = 0.57)*
0.5-2.9	144/150	1.00 (1.00 to 1.00)	1.00 (1.00 to 1.00)
3.0-4.9	164/133	1.28 (0.93 to 1.77)	1.25 (0.88 to 1.76)
5.0-6.9	112/94	1.26 (0.88 to 1.81)	1.25 (0.83 to 1.86)
7.0-9.9	102/85	1.26 (0.87 to 1.82)	1.34 (0.88 to 2.05)
10+	41/31	1.38 (0.81 to 2.34)	1.50 (0.82 to 2.76)
**(e) DCIS size (mm)**		(p_{trend} < 0.001)	(p_{trend} = 0.21)
<=10	238/156	1.00 (1.00 to 1.00)	1.00 (1.00 to 1.00)
11-20	182/165	0.73 (0.53 to 0.99)	0.84 (0.60 to 1.17)
21-50	120/129	0.61 (0.44 to 0.86)	0.87 (0.59 to 1.27)
51+	23/45	0.34 (0.19 to 0.61)	0.67 (0.35 to 1.28)
**(f) DCIS grade**		(p_{trend} = 0.44)	(p_{trend} = 0.17)
Low/intermediate	249/207	1.00 (1.00 to 1.00)	1.00 (1.00 to 1.00)
High	313/287	0.91 (0.71 to 1.16)	1.22 (0.92 to 1.61)
**(g) Laterality of DCIS**		(p_{trend} = 0.83)	(p_{trend} = 0.56)
Left	265/229	1.00 (1.00 to 1.00)	1.00 (1.00 to 1.00)
Right	297/264	0.97 (0.76 to 1.24)	0.93 (0.72 to 1.20)
**(h) Final margin distance (mm)**		($p_{heterogeneity}$ = 0.12)†	($p_{heterogeneity}$ = 0.36)†
5+	86/100	1.00 (1.00 to 1.00)	1.00 (1.00 to 1.00)
3-4	38/21	2.03 (1.00 to 4.12)	1.75 (0.85 to 3.61)
1-2	65/44	1.67 (0.96 to 2.91)	1.50 (0.83 to 2.71)
Involved	20/13	1.67 (0.68 to 4.08)	1.47 (0.58 to 3.74)
Before 2007	355/313	1.32 (0.92 to 1.88)	1.23 (0.76 to 1.99)
**(i) Treatment**		($p_{heterogeneity}$ < 0.001)	($p_{heterogeneity}$ < 0.001)
BCS + RT	66/75	1.00 (1.00 to 1.00)	1.00 (1.00 to 1.00)
BCS - RT	398/238	1.91 (1.32 to 2.76)	1.93 (1.23 to 3.02)
Mastectomy	100/180	0.64 (0.42 to 0.96)	0.66 (0.41 to 1.06)

BCS+RT: breast-conserving surgery, radiotherapy recorded
BCS-RT: breast-conserving surgery, radiotherapy not recorded
CI: confidence interval
P: results of tests for heterogeneity or trend
* Tests for trend excluding years 0.5-2.9: crude P=0.16; adjusted P=0.12
† Tests for trend across clear margin categories: crude P=0.05; adjusted P=0.15

Supplementary Figure S7. Comparison of the rates of ipsilateral and contralateral invasive breast cancer according to various factors in 24,779 women with unilateral screen-detected DCIS detected as a result of screening between April 2000 and March 2014 and who received surgery. Women recorded with oestrogen-receptor positive DCIS and receiving endocrine therapy are excluded. For each factor rates are shown relative to first category and adjustment is for all other factors except final margin distance. Final margin distance was not included in the adjustment as information on this variable was available only from 2007 onwards.

Supplementary Table S15. Characteristics of 29,044 women diagnosed with unilateral DCIS detected as a result of screening between April 2000 and March 2014 and who received surgery, according to oestrogen-receptor status and whether or not they were recorded as having received endocrine therapy.

	Oestrogen-receptor negative		Oestrogen-receptor negative	P for heterogeneity	Total
	No endocrine therapy	Endocrine therapy			
**Year of DCIS diagnosis**					
2000-04	3240 (51.7)	1355 (21.6)	1676 (26.7)	<0.001	6271 (100.0)
2005-09	6229 (59.8)	1856 (17.8)	2339 (22.4)		10,424 (100.0)
2010-14	9073 (73.5)	1054 (8.5)	2222 (18.0)		12,349 (100.0)
**Age at DCIS diagnosis (years)**					
<55	6296 (69.8)	1344 (14.9)	1377 (15.3)	<0.001	9017 (100.0)
55-59	3621 (59.9)	961 (15.9)	1467 (24.3)		6049 (100.0)
60-64	3818 (59.5)	970 (15.1)	1630 (25.4)		6418 (100.0)
65+	4798 (63.5)	992 (13.1)	1770 (23.4)		7560 (100.0)
**Region**					
Eastern	2595 (70.9)	241 (6.6)	824 (22.5)	<0.001	3660 (100.0)
North West	1961 (53.3)	1018 (27.7)	699 (19.0)		3678 (100.0)
Northern/Yorkshire	2798 (64.1)	521 (11.9)	1048 (24.0)		4367 (100.0)
Oxford	1092 (65.7)	156 (9.4)	415 (25.0)		1663 (100.0)
South West	3104 (66.0)	652 (13.9)	949 (20.2)		4705 (100.0)
Thames	3563 (62.7)	950 (16.7)	1166 (20.5)		5679 (100.0)
Trent	1519 (61.3)	440 (17.7)	520 (21.0)		2479 (100.0)
West Midlands	1917 (68.1)	289 (10.3)	607 (21.6)		2813 (100.0)
**DCIS size (mm)**					
<=10	6739 (70.7)	1425 (15.0)	1365 (14.3)	<0.001	9529 (100.0)
11-20	5335 (61.6)	1383 (16.0)	1939 (22.4)		8657 (100.0)
21-50	5141 (59.5)	1189 (13.8)	2311 (26.7)		8641 (100.0)
51+	1333 (60.2)	262 (11.8)	621 (28.0)		2216 (100.0)
**DCIS grade**					
Low/intermediate	8925 (76.1)	1882 (16.0)	927 (7.9)	<0.001	11,734 (100.0)
High	9609 (55.5)	2384 (13.8)	5319 (30.7)		17,312 (100.0)
**Laterality of DCIS**					
Left	9537 (63.6)	2222 (14.8)	3229 (21.5)	0.75	14,988 (100.0)
Right	8993 (64.0)	2043 (14.5)	3020 (21.5)		14,056 (100.0)
**Final margin distance (mm)**					
5+	7481 (69.3)	1126 (10.4)	2191 (20.3)	<0.001	10798 (100.0)
3-4	2090 (69.7)	363 (12.1)	544 (18.2)		2997 (100.0)
1-2	2932 (67.9)	598 (13.8)	790 (18.3)		4320 (100.0)
Involved	437 (68.6)	79 (12.4)	121 (19.0)		637 (100.0)
Before 2007	5602 (54.4)	2105 (20.5)	2585 (25.1)		10,292 (100.0)
**Surgery and radiotherapy**					
BCS + RT	3579 (66.7)	569 (10.6)	1220 (22.7)		5368 (100.0)
BCS - RT	9948 (65.5)	2747 (18.1)	2493 (16.4)		15,188 (100.0)
Mastectomy	5000 (58.9)	951 (11.2)	2537 (29.9)		8488 (100.0)
**Duration of follow-up (years)**					
0-4	9327 (72.8)	1142 (8.9)	2339 (18.3)		12808 (100.0)
5-9	6191 (59.4)	1877 (18.0)	2360 (22.6)		10428 (100.0)
11-14	3020 (52.0)	1246 (21.5)	1542 (26.5)		5808 (100.0)
**Laterality of IBC**					
Ipsilateral	398 (61.6)	82 (12.7)	166 (25.7)		646 (100.0)
Contralateral	337 (57.4)	94 (16.0)	156 (26.6)		587 (100.0)
Unknown	41 (49.4)	20 (24.1)	22 (26.5)		83 (100.0)
**Vital status on 31 Dec 2014**					
Alive	17,856 (64.2)	4009 (14.4)	5930 (21.3)		27,795 (100.0)
Emigrated	41 (55.4)	17 (23.0)	16 (21.6)		74 (100.0)
Dead	629 (53.5)	240 (20.4)	306 (26.0)		1175 (100.0)
**Cause of death**					
Breast cancer	65 (47.8)	29 (21.3)	42 (30.9)		136 (100.0)
Other causes	552 (54.1)	210 (20.6)	259 (25.4)		1021 (100.0)
Unknown cause	12 (66.7)	1 (5.6)	5 (27.8)		18 (100.0)
**Total**	18,542 (63.8)	4265 (14.7)	6237 (21.5)		29,044(100.0)

BCS+RT: breast-conserving surgery, radiotherapy recorded
BCS-RT: breast-conserving surgery, radiotherapy not recorded

	Cases/ Women	Ratio of rates (crude) 95% CI	Ratio of rates (adjusted) 95% CI
**(a) Ipsilateral**		($p_{heterogeneity}$ = 0.004)	($p_{heterogeneity}$ < 0.001)
ER+, no endocrine	398/18,542	1.00 (1.00 to 1.00)	1.00 (1.00 to 1.00)
ER+, endocrine	82/4265	0.64 (0.50 to 0.81)	0.62 (0.49 to 0.80)
ER-	166/6237	1.01 (0.80 to 1.28)	1.16 (0.90 to 1.50)
**(b) Contralateral**		($p_{heterogeneity}$ = 0.22)	($p_{heterogeneity}$ = 0.28)
ER+, no endocrine	337/18,542	1.00 (1.00 to 1.00)	1.00 (1.00 to 1.00)
ER+, endocrine	94/4265	0.85 (0.67 to 1.08)	0.87 (0.68 to 1.11)
ER-	156/6237	1.12 (0.86 to 1.46)	1.13 (0.85 to 1.52)
**(c) All**		($p_{heterogeneity}$ = 0.009)	($p_{heterogeneity}$ = 0.003)
ER+, no endocrine	776/18,542	1.00 (1.00 to 1.00)	1.00 (1.00 to 1.00)
ER+, endocrine	196/4265	0.77 (0.65 to 0.90)	0.76 (0.64 to 0.90)
ER-	344/6237	1.08 (0.89 to 1.29)	1.14 (0.93 to 1.40)

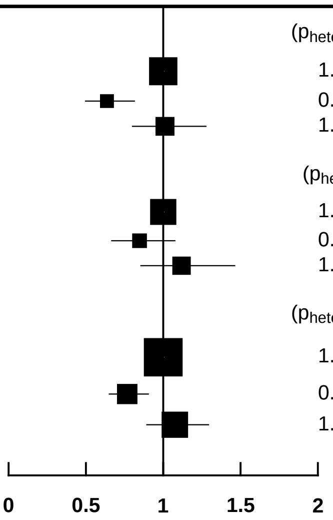

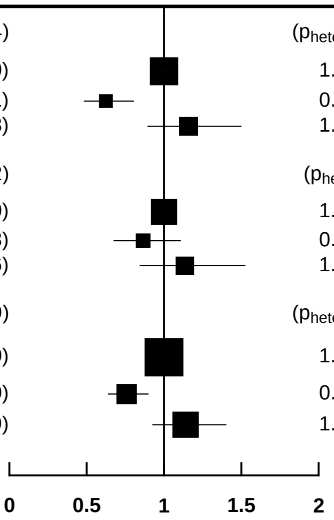

■ 95% CI

ER+, no endocrine: oestrogen-receptor positive DCIS, endocrine therapy not recorded
ER+, endocrine: oestrogen-receptor positive DCIS, endocrine therapy recorded
ER-: oestrogen-receptor negative DCIS
CI: confidence interval
P: results of tests for heterogeneity

Supplementary Figure S8. Incidence of ipsilateral and contralateral invasive breast cancer in 29,044 women with unilateral screen-detected DCIS according to oestrogen-receptor status and endocrine therapy. Rates are shown relative to first category in each section. Adjustment is for year of diagnosis, age at diagnosis, time since diagnosis, tumour size, DCIS grade, laterality and category of surgery/ radiotherapy.

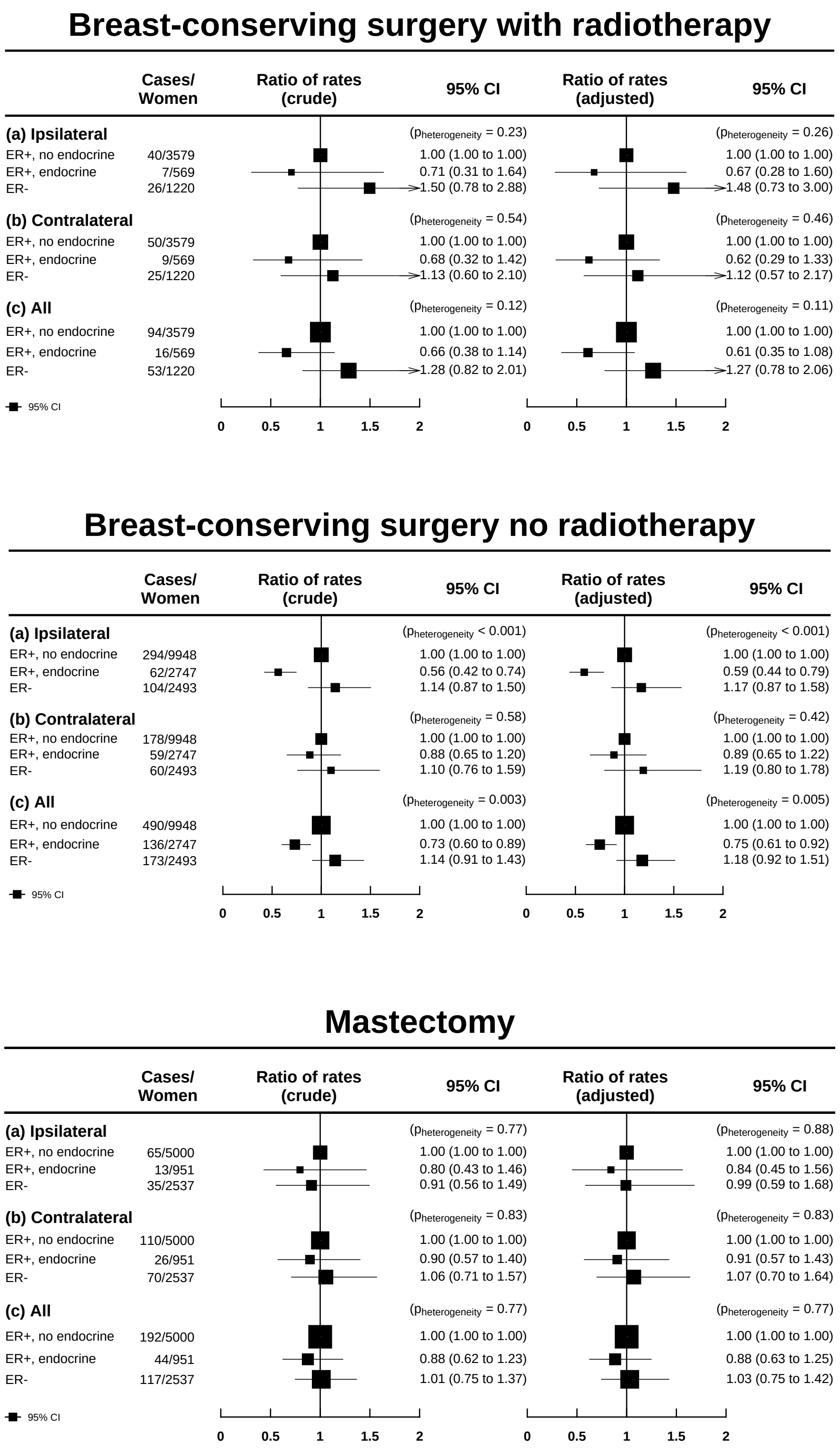

ER+, no endocrine: oestrogen-receptor positive DCIS, endocrine therapy not recorded
ER+, endocrine: oestrogen-receptor positive DCIS, endocrine therapy recorded
ER-: oestrogen-receptor negative DCIS
CI: confidence interval
P: results of tests for heterogeneity

Supplementary Figure S9. Incidence of ipsilateral and contralateral invasive breast cancer in 29,044 women with unilateral screen-detected DCIS who underwent surgery according to oestrogen-receptor status, endocrine therapy, and whether they received mastectomy, breast conserving surgery with radiotherapy, or breast conserving surgery without radiotherapy. Rates are shown relative to first category in each section. Adjustment is for year of diagnosis, age at diagnosis, time since diagnosis, tumour size, and DCIS grade and laterality.
